# Prognostic patterns in invasion lymph nodes of lung adenocarcinoma reveal distinct tumor microenvironments

**DOI:** 10.1038/s41698-024-00639-1

**Published:** 2024-07-30

**Authors:** Shen Lao, Zisheng Chen, Wei Wang, Yongmei Zheng, Shan Xiong, Ping He, Huan Yi, Jianfu Li, Feng Li, Shuting Li, Miao He, Xiaoyan Liu, Chuang Qi, Jianxing He, Wenhua Liang

**Affiliations:** 1 Department of Thoracic Surgery and Oncology, The First Affiliated Hospital of Guangzhou Medical University, State Key Laboratory of Respiratory Disease and National Clinical Research Center for Respiratory Disease, Guangzhou Institute of Respiratory Healthy, Guangzhou, China; 2 Department of Respiratory and Critical Care Medicine, the Affiliated Qingyuan Hospital, Guangzhou Medical University, Qingyuan People’s Hospital, Qingyuan, 511518 China; 3 Department of Pathology, The First Affiliated Hospital of Guangzhou Medical University, Guangzhou, China; 4 The State Key Laboratory of Translational Medicine and Innovative Drug Development, Jiangsu Simcere Diagnostics Co., Ltd, Nanjing, China

**Keywords:** Cancer, Surgical oncology

## Abstract

Tumor-draining lymph nodes (TDLNs) are usually the first station of tumor metastasis in lung cancer. TDLNs+ have distinct pathomorphologic and tumor microenvironment (TME)-compositional patterns, which still need to be thoroughly investigated in lung adenocarcinoma (LUAD). Here, we enrolled 312 LUAD patients with TDLNs+ from our institution between 2015 and 2019. 3DHISTECH was used to scan all of the TDLNs+. Based on morphologic features, TDLNs+ patterns were classified as polarized-type or scattered-type, and TME-compositional patterns were classified as colloid-type, necrosis-type, specific-type, and common-type. Multivariate analysis revealed an increased risk of early recurrence associated with scattered-type (HR 2.37, 95% CI: 1.06–5.28), colloid-type (HR 1.95, 95% CI: 1.03–3.67), and necrosis-type (HR 2.21, 95% CI: 1.13–4.89). NanoString transcriptional analysis revealed an immunosuppression and vascular invasion hallmark in scattered and necrosis patterns and an immunoactivated hallmark in polarized and common patterns. According to imaging mass cytometry (IMC), the scattered and necrosis patterns revealed that germinal centers (GC) were compromised, GCB cell and T cell proliferation were deficient, tumor cells had the potential for proliferation, and the immune attack may be weaker. In this study, we present evidence that LUAD patients have distinct patterns and immune hallmarks of TDLNs+ related to their prognosis.

## Introduction

Lung adenocarcinoma (LUAD) is the most prevalent subtype of lung cancer and has been associated with considerable prognostic heterogeneity^1–4^. Tumor-draining lymph nodes (TDLNs) serve as critical primary sites for tumor antigen exposure, regulating and cross-priming the antitumor immune response, and serving as sites of T cell invigoration required for checkpoint blockade therapy^5,6^. The tumor-invaded TDLNs (TDLNs+) are also strongly thought to predict a poor clinical outcome^7^. Additionally, TDLNs+ were linked to poor pathologic responses in primary tumors and were predictive of rapid post-treatment tumor relapse after neoadjuvant immune checkpoint inhibitors (ICIs) therapy^8^. Moreover, non-tumor invaded TDLNs (TDLNs−) are enriched for tumor-specific PD-1+ T cells, abundant PD-1/PD-L1-interactions derived from conventional dendritic cells (cDCs) and tumor-specific PD-1+ T cells but not tumor, are correlate with early distant disease recurrence, and PD-L1 blockade on cDCs elicits effective antitumor immunity^9^. These findings suggest that the microenvironmental status of TDLNs is critical for maintaining anti-primary tumor immune response.

Based on morphologic features, TDLNs+ patterns were classified as polarized-type or scattered-type, and tumor microenvironment (TME)-compositional patterns were classified as colloid-type, necrosis-type, specific-type, and common-type. We hypothesized that these distinct pathomorphologic and TME-compositional patterns, which are associated with differences in immune contexture, would influence the disease-free survival (DFS) of previously untreated and resectable LUAD patients.

This study enrolled 312 LUAD patients with TDLNs+ from our institution between 2015 and 2019, and 1348 TDLNs+ were analyzed. We aimed to analyze these populations’ TDLNs+ histological features and immune compositions and their association with patient outcomes.

## Results

### Patient characteristics

Table 1 summarizes the baseline characteristics of the 312 patients enrolled in the study. The cohort comprised 161 male patients (52%) and 220 never-smokers (71%). The most prevalent type of somatic mutation detected was the epidermal growth factor receptor (EGFR) mutation, present in 151 patients (49%), followed by the fusion mutation, which was present in 41 patients (13%). KRAS mutation was recorded in 34 patients (11%), while 86 patients (27%) had a wild-type gene profile. An examination of the primary tumor site revealed that the acinar-predominant histological pattern was the most frequent subtype, present in 148 patients (47%), followed by the papillary-predominant pattern, present in 70 patients (22%); the solid-predominant pattern presents in 61 patients (20%), and the micropapillary-predominant pattern, present in 33 patients (11%). A similar pattern was observed in the examination of the TDLNs+ site, where the acinar-predominant pattern was the most common subtype, present in 118 patients (38%). The solid-predominant pattern was the second most frequent subtype, present in 96 patients (31%), followed by the papillary-predominant pattern, present in 75 patients (24%), and the micropapillary-predominant pattern, present in 23 patients (7%). Additionally, 106 patients (34%) had a lymph node ratio (LNR) exceeding 0.33, and 118 patients (37%) demonstrated evidence of vascular invasion.

**Table 1:** Patients’ clinical and pathological characteristics

Clinicopathological characteristics		*n* (%)
Gender	Male	161 (52)
	Female	151 (48)
Age	≤60	158 (51)
	>60	154 (49)
Smoke	Never	220 (71)
	Ever	92 (29)
Driver mutation	EGFR	151 (49)
	KRAS	34 (11)
	FUSION	41 (13)
	Wild-type	86 (27)
Tumor size	≤3 cm	160 (52)
	>3 cm	152 (48)
Tumor location	RUL	86 (28)
	RML	36 (11)
	RLL	56 (19)
	LUL	80 (24)
	LLL	54 (18)
Vascular invasion	Absent	194 (63)
	Present	118 (37)
LNR	≤0.33	206 (66)
	>0.33	106 (34)
Pathological N stage	N1	164 (53)
	N2	148 (47)
DFS status	Stable	133 (43)
	Relapse	79 (25)
	Loss to follow-up	100 (32)
Infiltrated pattern	Polarized	285 (91)
	Scattered	27 (9)
Compositional pattern	Common	140 (45)
	Colloid	111 (36)
	Necrosis	47 (15)
	Specific	14 (4)
Primary tumor WHO classification	Lepidic	0 (0)
	Acinar	148 (47)
	Papillary	70 (22)
	Solid	61 (20)
	Micropapillary	33 (11)
Metastatic LN WHO classification	Lepidic	0 (0)
	Acinar	118 (38)
	Papillary	75 (24)
	Solid	96 (31)
	Micropapillary	23 (7)

*RUL* right upper lung, *RML* right middle lung, *RLL* right lower lung, *LUL* left upper lung, *LLL* left lower lung, *DFS* disease-free survival, *LNR* lymph node ratio.

### Morphopathological Patterns of TDLNs+ and Distribution

In the cohort of 312 patients, 5619 lymph nodes were surgically excised and subjected to histopathological examination. The median number of nodes per patient was 16^10^, ranging from 1 to 52. Of these, 1348 TDLNs+ were evaluated for their tumor-infiltrated and TME-composition patterns. The median TDLNs+ per patient was 3, ranging from 1 to 33. The examination of the tumor-infiltrated pattern revealed that 285 patients (91% of TDLNs+) demonstrated the polarized-type pattern, while 27 patients (9% of TDLNs+) exhibited the scattered-type pattern. An analysis of the TME-composition pattern indicated that 140 patients (45% of TDLNs+) showed the common-type pattern, 111 patients (36% of TDLNs+) displayed the colloid-type pattern, 47 patients (15% of TDLNs+) demonstrated the necrosis-type pattern, and 14 patients (4% of TDLNs+) showed the specific-type pattern (Table 1).

### Association between TDLNs+ patterns and clinicopathological features

Table 2 shows that the tumor-infiltrated TDLNs+ pattern (Group 1) differed significantly by gender (${\rm{\chi }}$2 = 4.169, *p* = 0.041), smoking status (${\rm{\chi }}$2 = 7.110, *p* = 0.008), pStage N (${\rm{\chi }}$2 = 4.384, *p* = 0.036), LNR status (${\rm{\chi }}$2 = 11.073, *p* = 0.001), and the presence of vascular invasion (${\rm{\chi }}$2 = 3.953, *p* = 0.047). Notably, the scattered pattern was more frequently observed in males, >3.0 cm tumor size, those with pathological stage N2, and those with higher LNR values, and it was also more commonly associated with vascular invasion.

**Table 2:** Association between infiltrated metLN patterns and patients’ clinicopathological features

Clinicopathological variables		Case	Infiltrated pattern		χ2	*p*-value
			Polarized	Scattered		
Gender	Male	161	142	19	4.169	0.041^*^
	Female	151	143	8		
Age	≤60	158	142	16	0.878	0.349
	>60	154	143	11		
Smoke	Ever	92	78	14	7.110	0.008^*^
	Never	220	207	13		
Tumor size	≤3 cm	160	150	10	2.401	0.121
	>3 cm	152	135	17		
Tumor location	RUL	86	80	6	7.055	0.133
	RML	36	33	3		
	RLL	56	47	9		
	LUL	80	77	3		
	LLL	54	48	6		
Driver mutation	EGFR	151	141	10	4.525	0.210
	KRAS	34	29	5		
	FUSION	41	35	6		
	Wild-type	86	80	6		
Vascular invasion	Absent	194	182	12	3.953	0.047^*^
	Present	118	103	15		
pStage N	N1	164	155	9	4.384	0.036^*^
	N2	148	130	18		
Primary tumor pathological classification (WHO)	Acinar	148	137	11	0.890	0.828
	Papillary	70	64	6		
	Solid	61	55	6		
	Micropapillary	33	29	4		
Metastatic LN pathological classification (WHO)	Acinar	118	112	6	6.751	0.080
	Papillary	75	69	6		
	Solid	96	82	14		
	Micropapillary	23	22	1		
LNR status	Low (<0.33)	206	196	10	11.073	0.001^*^
	High (>0.33)	106	89	17		

^*^Statistical significance determined by χ2 (chi-square) test (*p* < 0.05).

As displayed in Table 3, there was a significant correlation between the TME-composition TDLNs+ pattern (Group 2) and several clinicopathological factors, including tumor location (χ2 = 26.003, *p* = 0.011), pStage N (χ2 = 8.488, *p* = 0.037), LNR status (χ2 = 18.365, *p* < 0.001), and LN WHO classification (χ2 = 21.210, *p* = 0.012). Our results suggest that the composition of the TME within the TDLNs+ is closely associated with various clinicopathological factors. Additionally, the tumor-infiltrated TDLNs+ pattern (Group 1) and TME-composition TDLNs+ pattern showed strong relationships with pathological N-staging or LNR status. However, no significant relationship was found between the two patterns (χ2 = 3.187, *p* = 0.364) and between the common driver mutations in TDLNs+ and each pattern (Supplementary Fig. 4A–C). These results suggest that the two groups of TDLNs+ patterns are independent of each other.

**Table 3:** Association between compositional TDLNs+ patterns and patients’ clinicopathological features

Clinicopathological variables		Case	Compositional pattern				χ^2^	*p*-value
			Colloid	Necrosis	Specific	Common		
Gender	Male	161	58	26	8	69	0.752	0.861
	Female	151	53	21	6	71		
Age	≤60	158	49	26	9	74	3.604	0.308
	>60	154	62	21	5	66		
Smoke	Ever	92	36	15	4	37	1.232	0.745
	Never	220	75	32	10	103		
Tumor size	≤3 cm	160	54	19	6	81	5.346	0.148
	>3 cm	152	57	28	8	59		
Tumor location	RUL	86	24	19	3	40	26.003	0.011^*^
	RML	36	17	4	1	14		
	RLL	56	17	2	3	34		
	LUL	80	34	14	7	25		
	LLL	54	19	8	0	27		
Driver mutation	EGFR	151	68	18	5	60	14.542	0.104
	KRAS	34	8	5	2	19		
	FUSION	41	10	6	2	23		
	Wild-type	86	25	18	5	38		
Vascular invasion	Absent	194	68	24	8	94	4.127	0.248
	Present	118	43	23	6	46		
pStage N	N1	164	61	17	5	81	8.488	0.037^*^
	N2	148	50	30	9	59		
Primary tumor WHO classification	Acinar	148	49	18	8	73	9.921	0.357
	Papillary	70	32	8	2	28		
	Solid	61	20	13	2	26		
	Micropapillary	33	10	8	2	13		
Metastatic LN WHO classification	Acinar	118	49	13	2	54	21.21	0.012*
	Papillary	75	18	12	2	43		
	Solid	96	37	19	9	31		
	Micropapillary	23	7	3	1	12		
LNR status	Low (<0.33)	206	67	24	6	109	18.365	<0.001^*^
	High (>0.33)	106	44	23	8	31		

^*^Statistical significance determined by χ^2^ (chi-square) test (*p* < 0.05).

### Survival analysis

**Fig. 1:**
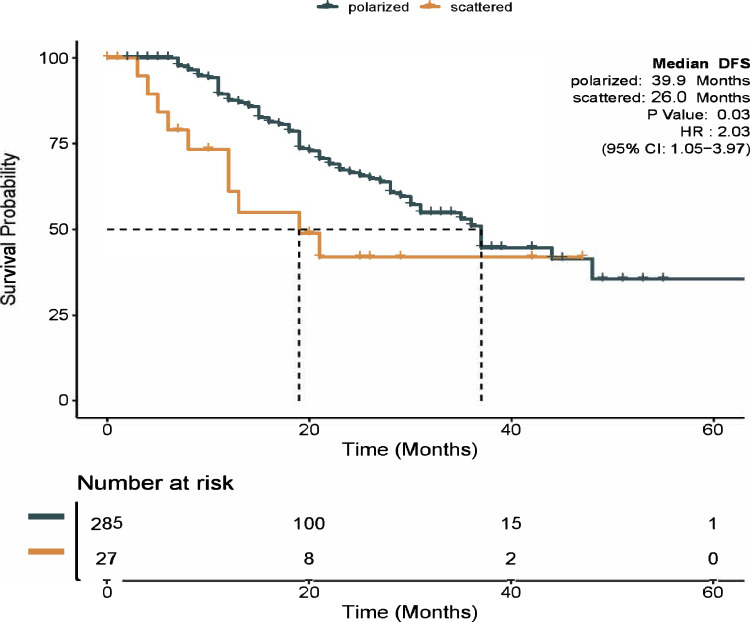
Kaplan–Meier survival curves for disease-free survival (DFS) according to the tumor-infiltrated pattern. The DFS for patients with the scattered-type TDLNs+ pattern was significantly lower than those with the polarized-type TDLNs+ pattern (26.0 months vs. 39.9 months, log-rank *p* = 0 .030).

In the cohort’s median follow-up period of 22.5 months (ranging from 3 to 66 months), 79 documented cases of recurrence were documented (with 32% censored). Analysis of the tumor-infiltrated TDLNs+ pattern revealed that patients with the scattered-type pattern experienced worse disease-free survival (DFS) than those with the polarized-type pattern (26.0 months vs. 39. 9 months, respectively; log-rank *p* = 0.030; Fig. 1).

**Fig. 2:**
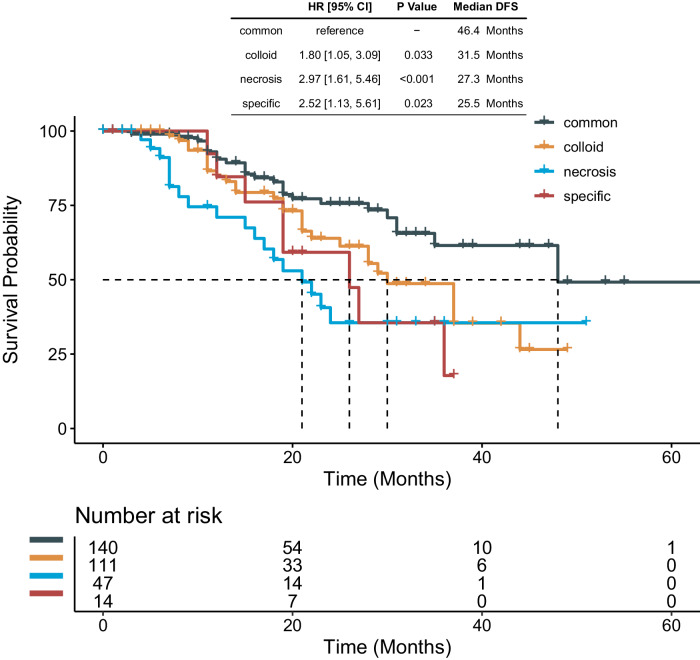
Kaplan–Meier survival curves for disease-free survival (DFS) according to the TME composition pattern. The DFS gradually decreased and was substantially poorer in patients with colloid- (31.5 months), necrosis- (27.3 months), and specific-type (25.5 months) TDLNs+ patterns than in individuals with common-type patterns (46.4 months).

Regarding the TME-composition TDLNs+ pattern, a significant correlation was found with DFS. The results showed that the common type was associated with the most favorable outcome with a median DFS of 46.4 months, followed by the colloid type (31.5 months), the necrosis type (27.3 months), and the specific type (25.5 months as depicted in Fig. 2). However, no significant correlation was identified between the WHO classification and recurrence (log-rank *p* = 0.081) (data not shown).

### Univariable and multivariable analysis

The results of the univariate and multivariable analyses are presented in Table 4. In terms of univariate analysis, conventional pathological factors, including tumor size >3.0 cm (HR = 1.59, *p* = 0.041), the presence of vascular invasion (HR = 1.90, *p* = 0.005), pStage N2 (HR = 2.04, *p* = 0.002), LNR high (HR = 2.43, *p* < 0.001), and WHO classification of the primary site, such as the solid-predominant type (HR = 1.91, *p* = 0.027) and micropapillary-predominant type (HR = 2.31, *p* = 0.007), were identified as being associated with poor DFS. Interestingly, the infiltrated pattern scattered-type (HR = 2.03, *p* = 0.036), and compositional patterns such as colloid-type (HR = 1.78, *p* = 0.036), necrosis-type (HR = 2.94, *p* < 0.001), and specific-type (HR = 2.48, *p* = 0.025) were found to be strongly linked to poor DFS.

**Table 4:** Univariable and multivariable analysis for variables affecting disease-free survival

		Univariate		Multivariate	
Clinicopathological variables		HR	*p*-value	HR	*p*-value
Gender	Male	Ref.		Ref.	
	Female	0.89	0.622	1.40	0.324
Age	≤60	Ref.		Ref.	
	>60	0.85	0.503	0.71	0.180
Smoke	Never	Ref.		Ref.	
	Ever	1.49	0.086	1.72	0.158
Tumor size	≤3 cm	Ref.		Ref.	
	>3 cm	1.59	0.041*	1.24	0.380
Tumor location	LLL	Ref.		Ref.	
	RUL	1.61	0.172	1.82	0.134
	RML	1.36	0.471	1.75	0.233
	RLL	0.65	0.333	0.62	0.305
	LUL	0.74	0.460	0.56	0.212
Driver mutation	EGFR	Ref.		Ref.	
	KRAS	1.04	0.911	1.09	0.857
	FUSION	1.07	0.840	1.62	0.236
	WT	1.14	0.611	1.26	0.441
Vascular invasion	Absent	Ref.		Ref.	
	Present	1.90	0.005^*^	1.79	0.030^*^
pStage *N*	N1	Ref.		Ref.	
	N2	2.04	0.002^*^	1.73	0.047^*^
Infiltrated pattern	Polarized	Ref.		Ref.	
	Scattered	2.03	0.036^*^	2.37	0.035^*^
Compositional pattern	Common	Ref.		Ref.	
	Colloid	1.78	0.036^*^	1.95	0.038^*^
	Necrosis	2.94	<0.001^*^	2.21	0.020^*^
	Specific	2.48	0.025^*^	1.97	0.142
Primary tumor pathological classification (WHO)	Acinar	Ref.		Ref.	
	Papillary	1.00	0.980	0.81	0.578
	Solid	1.91	0.027*	1.36	0.362
	Micropapillary	2.31	0.007*	1.98	0.071
Metastatic LN pathological classification (WHO)	Acinar	Ref.		Ref.	
	Papillary	0.82	0.570	0.92	0.836
	Solid	1.65	0.053	1.01	0.958
	Micropapillary	1.21	0.668	1.93	0.195
LNR status	Low (<0.33)	Ref.		Ref.	
	High (>0.33)	2.43	<0.001^*^	1.84	0.028^*^

*HR* hazard ratio, *Ref.* reference.

*Statistically significant category.

**Fig. 3:**
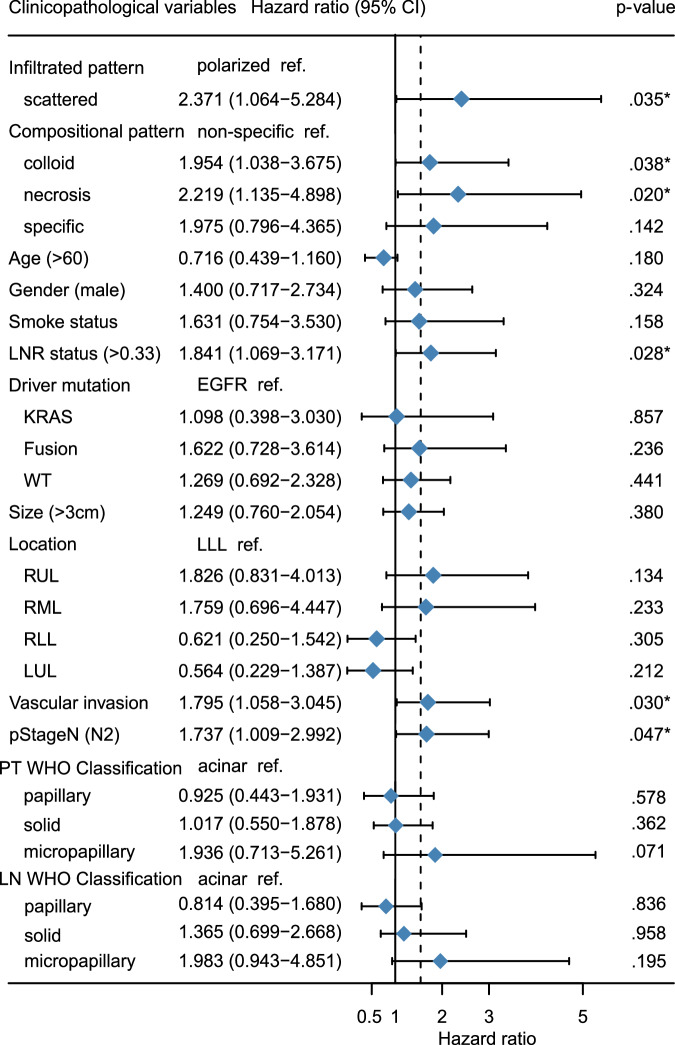
Forest plot of the risk factors for DFS of LUAD by univariable analysis. The Hazard ratio of DFS and 95% confidence intervals were reported.

Additionally, our results indicate that the scattered-type (HR = 2.37, 95% CI: 1.06–5.28, *p* = 0.035), colloid-type (HR = 1.95, 95% CI: 1.03–3.67, *p* = 0.038), and necrosis-type (HR = 2.21, 95% CI: 1.13–4.89, *p* = 0.020) remained significant in the multivariable analysis, suggesting that they may serve as independent predictive factors for poor DFS (Fig. 3). Furthermore, our analysis highlights the continued significance of classic risk factors such as LNR high (HR = 1.84, 95% CI: 1.06–3.17, *p* = 0.028), present vascular invasion (HR = 1.79, 95% CI:1.05–3.04, *p* = 0.030), and pStage N2 (HR = 1.73, 95% CI:1.00–2.99, *p* = 0.047) in determining DFS outcomes.

### Transcriptional analysis of distinct TDLNs+ pattern

**Fig. 4:**
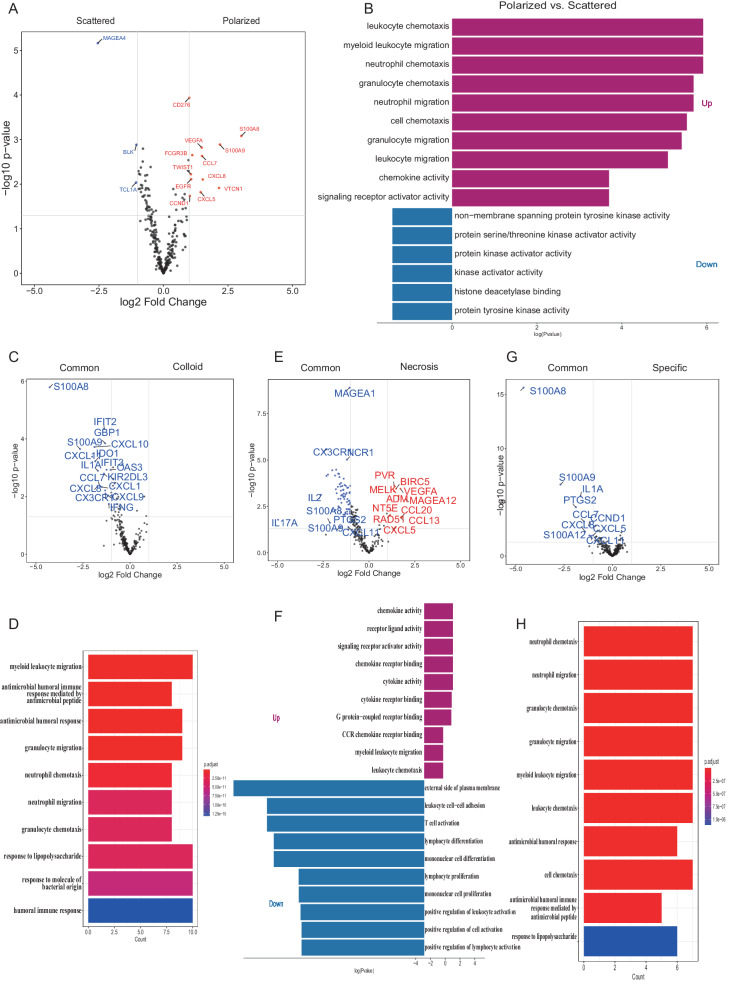
Transcriptional analysis of distinct TDLNs+ pattern. The NanoString nCounter 289 panel performed RNA sequencing. **A** Volcano plot of polarized pattern vs. scattered pattern. **B** GO-BP terminology enrichment analysis related to polarized pattern vs. scattered pattern up and down-regulated genes. **C**, **E**, **G** Volcano plot of colloid pattern vs. common pattern, necrosis pattern vs. common pattern, and specific pattern vs. common pattern, and **D**, **F**, **H** GO-BP terminology enrichment analysis related to colloid pattern vs. common pattern, necrosis pattern vs. common pattern, and specific pattern vs. common pattern up and down-regulated genes, respectively.

To comprehensively evaluate the immune components of TDLNs+ patterns, we performed mRNA profiling analysis using the NanoString nCounter 289 Panel. Our results showed that MAGEA4, BLK, and TCL1A were highly expressed in the scattered pattern, while CCL7, CXCL5, CXCL8, CD276, EGFR, CCND1, TWIST1, and S100A8 were highly expressed in the polarized pattern (Fig. 4A). Additionally, we observed that protein kinase activator activity and protein serine/threonine kinase activator activity were enriched in significant down-regulated genes of the polarized pattern, which contributed to NSCLC survival and resistance to chemotherapy and radiation^11^. While myeloid leukocyte migration, neutrophil chemotaxis, and neutrophil migration were enriched in significantly upregulated genes of the polarized pattern (Fig. 4B), which linked to tumor proliferation and inflammatory mediator synthesis^12^ and neutrophils recruited by chemotaxis may turn into tissue resident neutrophils associated with anti-PD-L1 treatment failure^13^. Further analysis of the TME-composition TDLNs+ pattern (compared with the common pattern) revealed that CXCL1, CXCL8, CXCL9, CXCL10, CXCL11, IDO1, and IFNG were significantly down-regulated in the colloid pattern, IL2, IL17A, CXCL11, PTGS2, and CX3CR1 were significantly down-regulated in the necrosis pattern, and IL1A, CXCL5, CXCL8, CCL7, and CCND1 were significantly down-regulated in the specific pattern. GO-BP terminology enrichment analysis revealed that the significantly down-regulated genes of the colloid pattern were associated with myeloid leukocyte migration, granulocyte migration, and neutrophil chemotaxis. The significantly upregulated genes of the necrosis pattern were enriched in myeloid leukocyte migration, leukocyte chemotaxis, and chemokine activity, etc., while the significantly down-regulated genes were involved in T cell activation, lymphocyte differentiation and proliferation, etc. Furthermore, the significantly down-regulated genes of the specific pattern were involved in neutrophil chemotaxis, neutrophil migration, and granulocyte chemotaxis (Fig. 4C–H).

**Fig. 5:**
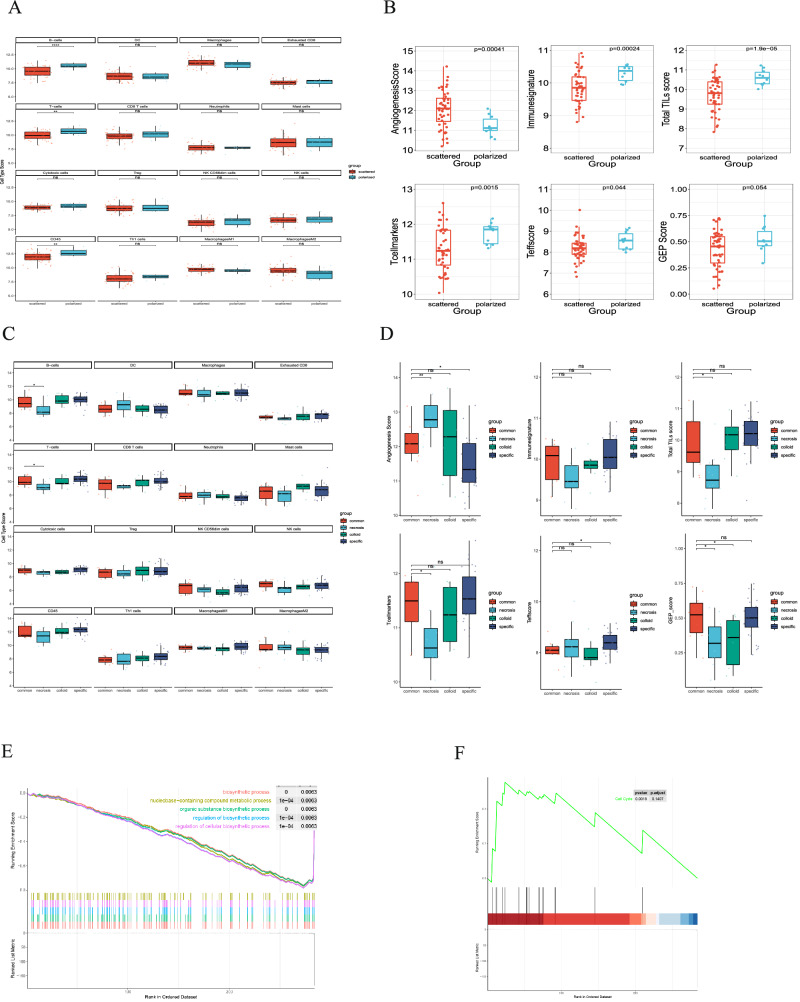
Transcriptional analysis of distinct TDLNs+ pattern based on signature score. **A** Comparison of the immune cell type score between polarized and scattered patterns. **B** Comparison of the immune signature between the polarized and scattered pattern. **C** Comparison of the immune cell type score between common, necrosis, colloid, and specific patterns. **D** Comparison of the immune signature between common, necrosis, colloid, and specific patterns. **E** GSEA BP plot with top 5 enrichment pathways between necrosis and common pattern. **F** The GSEA Reactome enrichment of cell cycle pathways between necrosis and common pattern.

Immune activation gene-expression signature defines a distinct subtype in the polarized pattern, a greater immune infiltration, characterized by elevated levels of CD45 immune cells, including T-cells and B-cells, was observed in the polarized pattern within the TDLNs+ group as demonstrated by the elevated cell-type score (Fig. 5A). Furthermore, a positive correlation was found between a higher angiogenesis score and the scattered pattern, while a lower immune score was noted for several immune-related parameters, including total TILs, T-cell markers, and T-effector score (Fig. 5B). In the TME-composition TDLNs+ pattern, a decreased level of immune cell infiltration was observed in the necrosis subtype compare with the common pattern, including T cells, B cells, NK cells, Th1 cells, and CD8+ T cells, however, immunosuppressive DC cells and macrophage M2 cells were high in the necrosis pattern (Fig. 5C). This necrosis pattern was also positively associated with a high angiogenesis score, a lower immune signature scores include TILs score, T cell marker score, and GEP score (Fig. 5D). The GSEA results revealed prominent enrichment in signatures related to the biosynthetic process and metabolic process, which downregulated in the necrosis pattern group, whereas cell cycle pathway was upregulated in the necrosis pattern group (Fig. 5E, F).

### IMC-based protein profiling of distinct TDLNs+ pattern

Previous evidence suggests that TDLNs+ are reduced pathologic complete or major response in the paired primary tumor and was associated with rapid post-treatment tumor relapse^8^. To further profile cellular phenotype and spatial organization of the TDLNs+ patterns with distinct clinical outcomes, we employed a 35-marker IMC panel on the corresponding FFPE section. Using IMC data, we generated a map of TME heterogeneity. As for the polarized, common, and colloid patterns, which are associated with relatively good DFS, the GC was complete and mature, expressing CD20, CD21, CD4, and CCR7, with visible FAP+ fibroblastic reticular cell (FRC) network structure and Ki-67+ CD20+ GCB cells, and Ki-67+ T cells, CCL21 was mainly distributed in a regular CD4+ T and CD8+ T cell interaction network outside GC. The microenvironment for progenitor T cells is provided by regular T and B lymphocyte networks^14^. Tumor cells failed to express or had a minimal expression of Ki-67 and showed strong signs of immune attack through the presence of IFN-γ, especially in the polarized pattern.

**Fig. 6:**
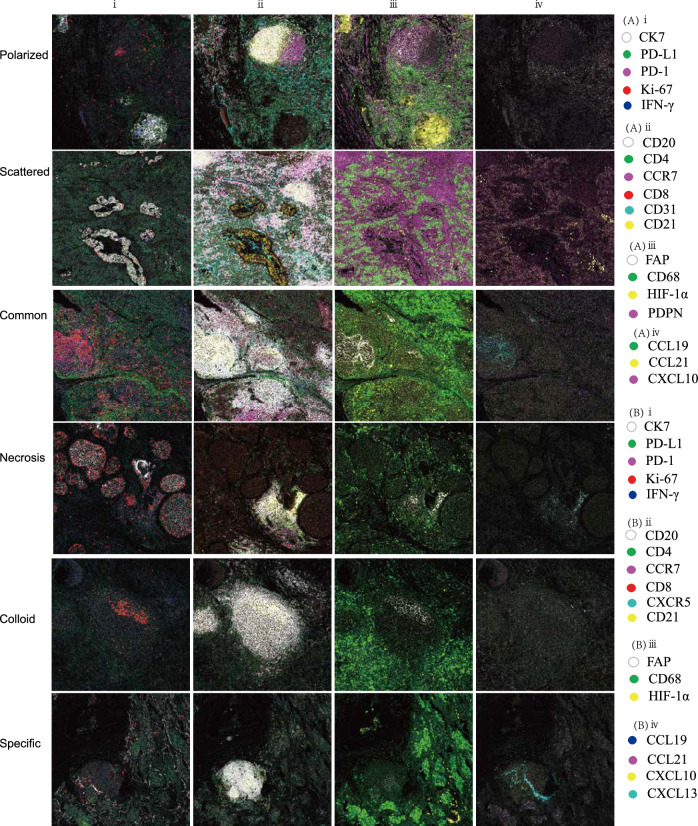
IMC-based protein profiling of distinct TDLNs+ pattern. i. Epithelial marker, ii. Mature GC marker, iii. Stromal and macrophage marker, iv. Chemokine marker. **A** Polarized pattern and scattered pattern. **B** Common, necrosis, colloid, and specific pattern.

Additionally, the vascular structure was complete, with antigen-specific PD-1+ CD8+ T cells visible within the CD31+ vasculature (the polarized pattern). However, the scattered, necrosis, and specific patterns positively correlated with shorter DFS. Common features include tumor cell expressed Ki-67 (especially in the necrosis pattern), weak signs of immune attack through the absence of IFN-γ, and low PD-L1 expression; moreover, FAP + FRC network and GC structure were destroyed, Ki-67+ CD20+ GCB cells, Ki-67+ T cells were absent, with no antigen-specific PD-1+ CD8+ T cells enrichment, and an irregular interaction network was seen between CD4+ T and CD8+ T cells outside GC; furthermore, disorderly arrangement of PDPN+ fibroblasts and CD68+macrophages was observed (especially in the scattered pattern), and vasculature was deformed and irregular (especially in the scattered pattern), which consistent with what NanoString panel observed (Figs. 5B, D and 6A, B).

Moreover, we sought molecular discrepancy for TDLNs+ patterns by a hierarchical consensus meta-clustering algorithm FlowSOM (v1.18.0). We identified 21 meta-clusters in the infiltrated TDLNs+ pattern and 16 meta-clusters in the TME-compositional TDLNs+ pattern (Supplementary Fig. 5A–G, Supplementary Fig. 6A–F). Of them, we found that the scattered pattern predominantly constrains meta-cluster 8 (HLA_ABC^lo^CD20^lo^ CD21^lo^), and meta-cluster 18 (PDPN^hi^CD31^hi^), as shown in Supplementary Fig. 5H, I). In terms of the compositional pattern, we observed a high cellular density of meta-cluster 8 (PDPN^hi^CD31^hi^) and meta-cluster 14 (CK7^hi^CD44^hi^HLA_ABC^lo^), see Supplementary Fig. 6G, H) within the necrosis pattern, while a group of suspected meta-cluster 1 anergic antigen-presenting cells (CD63/Lamp3^hi^CD68^lo^HLA_ABC^lo^), see Supplementary Fig. 6I) was found in the colloid subtype. By contrast, the common pattern was detected with larger densities of activated immune cells, meta-cluster 9 (HLA_ABC^hi^CD3^hi^CD4^hi^CD68^hi^CD107a^hi^), see Supplementary Fig. 6J).

## Discussion

Our study is interesting in that it classifies the TME of TDLNs+ based on immune panel transcriptional and IMC-based protein profiling. Firstly, in our cohort of 312 patients, 5619 lymph nodes were surgically excised and subjected to histopathological examination, and 1348 TDLNs+ were evaluated for their tumor-infiltrated pattern and TME-composition pattern. The sample size of TDLNs+ is large enough and representative. We discover that polarized, common, and colloid patterns are associated with relatively longer DFS, whereas scattered, necrosis, and specific patterns are associated with shorter DFS. Interestingly, previous studies have shown a similar dispersed pattern in some invasive cancers such as breast lobular carcinoma^15–17^, pancreatic ductal adenocarcinoma^18^, and Merkel cell carcinoma^19^. For example, previous colorectal cancer studies classified the desmoplastic reaction as mature, intermediate, or immature based on the reactive fibrous zone containing keloid-like collagen and myxoid stroma. The results showed that the best long-term survival was seen in the mature group^20^, absent myxoid stroma and keloid-like collagen bundles (marked myxoid stroma, and marked keloid-like collagen bundles were set as references)^21^. TME analysis indicated that tumor-infiltrating lymphocytes were highest in the mature group^20,21^, which may represent the Infiltrated-inflamed phenotypes^22^.

Secondly, NanoString transcriptional sequencing discovered that immune cell signals such as CD45 cells, T cells and B cells, and immune signature include immune signature, Total TILs score, T cell markers, and Teff score are significantly higher, and angiogenesis score was significantly lower in polarized patterns than in scattered patterns. Similar phenomena exist in common and necrosis patterns. Third, a 35-marker IMC panel verified from protein levels and found that the GC of the polarized, common, and colloid patterns are fully developed, and GCB and T cells are proliferating. Furthermore, outside of the GC, CD4+ T and CD8+ T cells interact with one another as network rules. However, scattered, necrosis, and specific patterns revealed that GC was compromised, GCB and T cell proliferation was deficient, interaction networks of CD4+ T and CD8+ T outside GC vanished, tumor cells had the potential for proliferation, and the immune attack was weak, which may indicate progenitor T cells inability to reside TDLNs+ effectively or dysfunction.

Furthermore, the lymph node GC and mature TLS of solid tumors are organized in a network of CD3, CD4, CD8, CD20, and CD21^23,24^. We found that the scattered pattern predominantly constrains HLA_ABC^lo^CD20^lo^ CD21^lo^ and PDPN^hi^CD31^hi^ (lymphatic endothelial cells, PDPN+ CD31+) (Supplementary Fig. 5H, I), which is consistent with the transcriptional sequencing, that is, Immunesignature, Total TILs score, Tcellmarkers, and Teffscore are significantly lower, and Angiogenesis Score was significantly higher in scattered pattern. A previous breast cancer study showed that PDPN+ macrophages also localize to the proximity of tumor lymphatics and induce lymphangiogenesis and lymphoinvasion^25^. As for the compositional pattern, we found a high cellular density of PDPN^hi^CD31^hi^ and CK7^hi^CD44^hi^HLA_ABC^lo^ (Supplementary Fig. 6G, H) within the necrosis pattern, CD63/Lamp3^hi^CD68^lo^HLA_ABC^lo^ in the colloid subtype (Supplementary Fig. 6I), and HLA_ABC^hi^CD3^hi^CD4^hi^CD68^hi^CD107a^hi^ within the common pattern (Supplementary Fig. 6J), which is also consistent with the transcriptional sequencing, that is, Angiogenesis Score was significantly higher in necrosis pattern, Immunesignature and GEP_score were higher in common pattern. Our recent LUAD study showed that CD44+ lung cancer stem cells promoted brain metastases via GPR124-enhanced trans-endothelial migration^26^.

A previous study reported that an exhausted phenotype of mixed subset CD8+ T cells emerged 72 h after they entered the primary tumor. CD8+ T cells with a memory or stem-like phenotype balanced by the traveling interaction between TDLN and the primary tumor to maintain antitumor immunity^27^. In early disease, TDLN- is essential for immunotherapeutic response and antitumor immune activation, but TDLN+ is not necessary for immunotherapeutic response in advanced disease; tumor antigen-specific CD8+ T cells are not able to survive in the TDLN+ microenvironment. Furthermore, at an advanced stage of tumor development, TDLN- transitions from an immunoactivated to a TDLN+ immune tolerance state, as evidenced by a significantly lower concentration of IFN-γ in TDLN+ than in TDLN−^28^. TDLN-enriched progenitor exhausted CD8+ T cells (Tpex); the proportion of Tpex decreased during immunotherapy but localized around TDLN-dendritic cells, proliferating into intermediate-exhausted CD8+ T cells. It has the function of differentiation and immune killing, but in TDLN+, the immunosuppressive microenvironment destroys these processes^29^. Furthermore, our recent study found that for those postresectional recurred NSCLC patients who planned for adjuvant immunotherapy, dissected lymph node count ≤16 is associated with immunotherapy prognostic benefit, and this may be related to the retention of more CD8+ central memory T cells^30^.

Inevitably, this work is limited by a small transcriptional sequencing and IMC sample size. Another limitation of the study is the need for more validation of the prognostic effect of the subgroups in an additional cohort. Moreover, it is known that immune recognition of tumors and their neoantigens is dependent both on the clonality of the neoantigens presented and on the diversity of the T cell repertoire. However, IMC cannot be used to analyze T cell repertoire, and we hope that NanoString transcriptional sequencing can still be carried out in the future to assess the results further.

In summary, our study examines clinical and immune characteristics in the TDLNs+ of LUAD. Polarized and common patterns are linked to longer DFS, with higher immune signatures but lower angiogenesis scores. The GC in these patterns is fully developed and proliferative, with rule interactions between CD4+ T and CD8+ T cells outside the GC and signs of immune attacking the tumor cells.

## Methods

### Patients

We conducted a retrospective study of 312 LUAD patients who underwent R0 resection between 2015 and 2019 at the First Affiliated Hospital of Guangzhou Medical University. Patients with non-tumor invaded TDLNs (TDLNs−) and missing data were excluded, as were those with late-stage IV (due to potential confounders). Clinicopathological data were retrieved from electronic records. The retrospective study was conducted at a single academic medical center (The First Affiliated Hospital of Guangzhou Medical University), approved by the Institutional Review Board (Medical Research Ethics Review 2021 No. 145), and conducted under the Declaration of Helsinki. This study is compliant with the ‘Guidance of the Ministry of Science and Technology (MOST) for the Review and Approval of Human Genetic Resources’. Institutional review board informed consent was waived because this study was retrospective.

### Pathology and histological evaluation of resected lymph nodes

A Pannoramic 250 Scanner (3DHISTECH, Hungary) was used to scan all available H&E-stained formalin-fixed paraffin-embedded (FFPE) slides of metastatic lymph nodes. Three pathologists (Y.M.Z., S.T.L., and P.H.) then reviewed the images using CaseViewer 2.2 (3DHISTECH, Hungary), with any discrepancies in their readings being discussed until a consensus was reached.

**Fig. 7:**
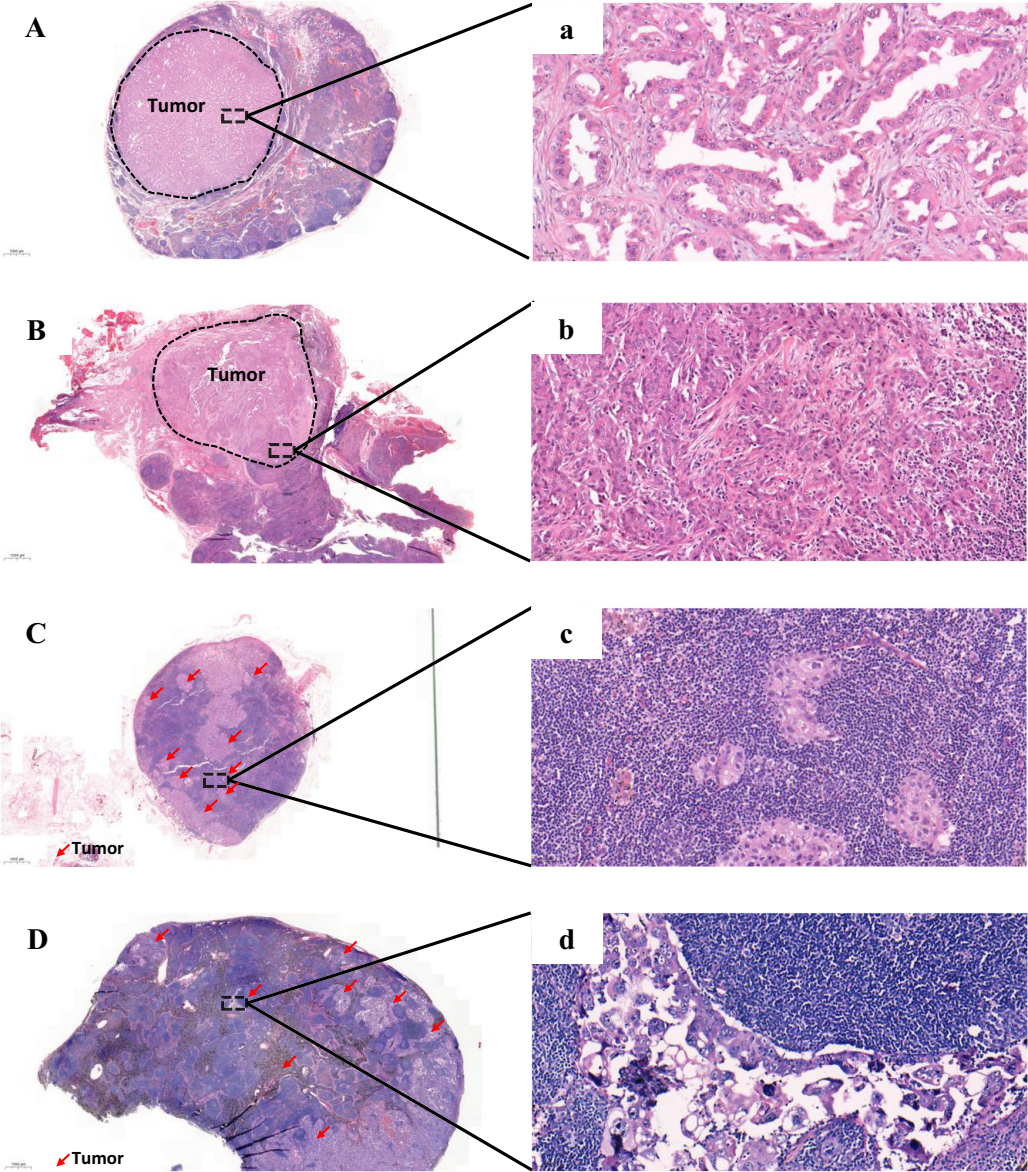
The tumor-infiltrated pattern in the TDLNs+ of LUAD. Polarized pattern: **A**, **B** (10x); a-b (200X). Metastatic cancer cells infiltrated the TDLNs+ as cohesive clusters and displayed a polarized distribution within the TDLNs+. Scattered pattern: **C**, **D** (10x); c-d (200X). Metastatic cancer cells infiltrated the TDLNs+ as isolated nests or clusters and were randomly distributed within the TDLNs+. Red arrow: tumor cluster.

**Fig. 8:**
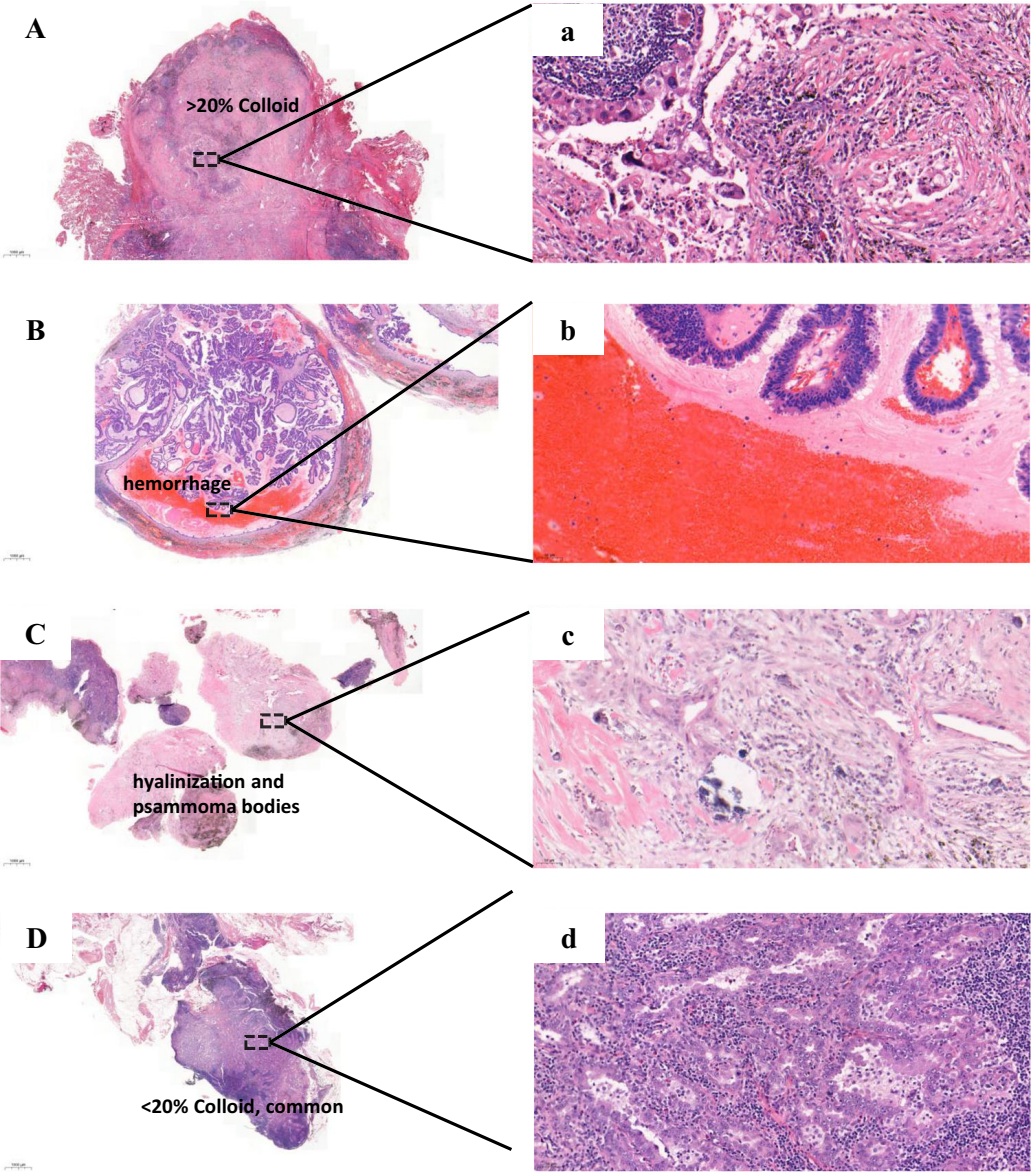
The TME-composition pattern in the TDLNs+ of LUAD. Colloid-type: **A** (10x), a (200X). A colloid component exceeding 20%; Necrosis-type: **B** (10x), b (200X). Characterized by the presence of necrotic cores derived from either the tumor or hemorrhage in a TDLN+; Specific-type: **C** (10x), c(200X). Displayed typical hyalinosis or psammoma bodies; Common-type: **D** (10x), d (200X). It exhibits a common appearance aspect with a poor collagen pool and lacks the presence of necrosis or specific features.

In addition to the well-described conventional histological patterns of LUAD primary tumor (including acinar-, papillary-, solid-, and micropapillary-predominant, as per the 2021 WHO classification)^31–34^, we identified several variations in the morphological features of TDLNs+. The optimal cut-off value has been determined on the basis of DFS by using an X-tile program (v 3.6.1). The X-tile statistically tests each division based on each cut-off point. Then, the 10% cut-off was calculated. Those TDLN+ without scattered-type appearance were excluded since the scattered ratio (SR) represents the ratio of scattered-type TDLNs+ to the total number of TDLNs+. These were classified into two distinct groups (as illustrated in Figs. 7 and 8):

——Group 1. We characterized the tumor-infiltrated pattern based on the distinctive distributions of metastatic tumors in a TDLN+. The pattern was further divided into two subtypes: 1) The polarized type—In this subtype, metastatic tumor cells infiltrated the TDLNs+ as cohesive clusters and displayed a polarized distribution within the TDLNs+ (as shown in Fig. 7A, B). 2) The scattered-type—In this subtype, metastatic tumor cells infiltrated the TDLNs+ as isolated nests or clusters and were randomly distributed within the TDLNs+ (as shown in Fig. 7C, D). To further characterize this pattern, we introduced the scattered ratio (SR) metric, which represents the ratio of scattered-type TDLNs+ to the total number of TDLNs+. Our categorical analysis using a cut-off point of 10% showed that patients with at least one scattered appearance in the examined TDLNs+ could be classified as having the scattered-type pattern. Therefore, patients with at least one scattered appearance in their examined TDLNs+ can be classified as having the scattered-type pattern (Supplementary Fig. 1A, B).

——Group 2. We defined the TME-composition pattern based on the heterogeneity in the composition of a TDLN+. The diverse morphological components within the TDLNs+ led us to classify it into the following subtypes (collagen-, necrosis-, specific-, and common-type): 1) The colloid-type—We define the colloid pattern as the presence of a specific quantity of collagen fibers within TDLN+. We then identified a variant characterized by a tumor environment that was predominantly composed of collagen, with a colloid component exceeding 20% (Fig. 8A, Supplementary Fig. 2A). The collagen component was recorded in 5% increments. To further stratify this pattern, we introduced a metric called the colloid ratio (CR) and determined 20% as the optimal cut-off point based on the disease-free survival (DFS) outcome; 2) The necrosis-type—This subgroup was characterized by the presence of necrotic cores derived from either the tumor or hemorrhage in a TDLN+, as depicted in Fig. 8B, Supplementary Fig. 2B, C; 3) The specific-type—where TDLNs+ displayed typical hyalinosis or psammoma bodies (Fig. 8C, Supplementary Fig. 2D, E). In particular instances, Hematoxylin and Eosin (H&E) staining reveals a consistent appearance with a notable affinity for Congo Red. These cases are systematically classified as a unique pattern identified as hyalinization (glassy change). This subset is situated within the broader category of collagenization, representing a distinctive pattern characterized by hyalinization. It is essential to highlight that morphological changes indicative of a glassy appearance may not accompany this colloid pattern. 4) The common-type—where TDLNs+ exhibited a common appearance aspect with a poor collagen pool and lacked the presence of necrosis or specific features (Fig. 8D, Supplementary Fig. 2F). Classifications are derived from expert pathologist consensus, ensuring accuracy through independent review and collective insight.

### Transcriptional analysis of the tumor-immune microenvironment

In this study, we comprehensively analyzed the tumor-immune microenvironment (TIME) using the various TIME signature profiling panels on TDLNs+ of LUAD patients. *N* = 10 for each group; however, in the RNA extraction process, the polarized pattern failed in 1 case, the colloid failed in 1 case, the specific pattern failed in 2 cases, and the common pattern failed in 2 cases (Supplementary Table 1). Gene expression was quantified through the utilization of the nCounter platform, which was developed by NanoString technologies (Seattle, WA). Transcriptional profiling relied upon a 289-immuno-gene panel specifically designed for investigating the immune response in cancer (Supplementary Table 2). With the aid of this comprehensive panel, one can concurrently evaluate a total of 289 genes associated with this immune response. Following that, multiple quality control indicators were identified for every sample. These encompassed the evaluation of imaging quality, the density of bindings, the linearity of positive controls, the limit of detection of positive controls, as well as the positive and content normalization factors. Samples eligible for quality control underwent subsequent analysis. To minimize technical variability in the assay, the raw data for each sample and gene were standardized against internal ERCC controls based on nSolver 2.6 software. Following this step, the counts were normalized by the geometric mean of endogenous housekeeping genes and then subjected to a log2 transformation.

Fourteen immune cell type marker genes were retrieved from published studies^35–37^, and Macrophages M1 and Macrophages M2 from previous reports. All TME cell infiltration scores were calculated as the arithmetic mean of the constituent genes^35^. The analysis was carried out using the nCounter system (NanoString Technologies, USA), and targeted RNA sequencing was performed on all 6 subgroups. To assess the immunophenotyping and immunotherapy efficacy, we employed previously reported analytical methods, including Total TILs^35^, Teffector^38^, Angiogenesis^39^, GEP gene signature score^40^, among others. These methods provide a comprehensive understanding of the interactions between the immune cells and the tumor in the microenvironment, which is critical for designing effective immunotherapy strategies.

Differential gene expression analysis was conducted using the NanoStringDiff package to identify differentially expressed genes (DEGs) across distinct groups. The criteria employed to ascertain DEGs comprised a significance level of *P* < 0.05 and an expression fold change (FC) ≥ 2 or FC ≤ 0.5. The Clusterprofiler^41^ was employed to conduct KEGG/GO enrichment analysis and Gene Set Enrichment Analysis. As an input file, the list of gene IDs was utilized. The Benjamini-Hochberg method was applied to refine the significance of *p*-values. A threshold of 0.05 was determined as the cut-off for *p*-values. In order to present the enrichment results, the ggplot2 was employed.

### The 35-antibodies imaging mass cytometry (IMC) panel

We designed an antibody panel to specifically target epitopes associated with LUAD, markers related to the germinal center, cytokines/chemokines & receptors, adhesion molecules, etc. The panel was also designed to distinguish between different cell types, such as epithelial, endothelial, mesenchymal, and immune cells (Supplementary Table 3, Supplementary Fig. 3).

### IMC panel staining and acquisition

The First Affiliated Hospital of Guangzhou Medical University fixed the tissue samples in formalin and embedded in paraffin. Tissue sections were stained using the antibody panel from Supplementary Fig. 3, then deparaffinized in xylene and rehydrated using a graded series of alcohols. Heat-induced epitope retrieval was performed in Tris-EDTA buffer, pH 9.0, using a 95 °C water bath for 20 min, and tissue sections were blocked with 3% BSA and 5% goat serum in TBS for 1 h. Samples were then incubated overnight at 4 °C in TBS, 0.1% Triton X-100 and 1% BSA in the primary antibody at optimal antibody dilution ratios based on preliminary experiments (1:50, 1:100, 1:200). Tissue samples were washed and dried, and IMC measurements were performed^42^. Autotuning of the Hyperion mass cytometry system (Fluidigm Corporation, South San Francisco, CA, USA) was accomplished by employing a 3-element tuning slide, adhering to the manufacturer’s stipulated tuning protocol. Regions of interest (ROIs) were handpicked after meticulous examination of sequential tissue sections that were stained with H&E. ROIs measuring 1000 × 1000 µm were acquired at 200 Hz for around 2 h. MCD files were exported and viewed using the Fluidigm MCDTM viewer. Each marker was visually inspected to optimize signal-to-noise separation, and a minimum signal threshold of one or two dual counts was established in the Fluidigm MCDTM viewer. A total of 208,483 cells were identified in 15 representative images (3 patients for each TDLN+ pattern).

### IMC data analysis

Data obtained from the Hyperion mass cytometry system were first converted to TIFF format using the Fluidigm MCDTM Viewer. The individual cells were segmented using CellProfiler (v.4.1.3), and a single-cell segmentation mask was exported. These segmentation masks were overlaid onto TIFF images of the 35 channels, and the mean expression levels of markers and spatial features of single cells were extracted using histoCAT (v1.7.6). The resulting data were exported as .fcs files for further analysis. The data were scaled with arcsinh-transformation and analyzed in R (v3.6.3). First, single alive cells were clustered using a deep-learning-based clustering algorithm, followed by Phenograph (v0.99.1) analysis. Rtsne (v.0.15) was used for high-dimensional data reduction in R. Once every single cell was assigned a cluster, the clusters were manually annotated based on their marker expression patterns compared to known immune cell types.

### Statistical analysis

The statistical analysis was performed using SPSS 26 (SPSS Inc.), R (v 3.6.3) and Prism 7 (GraphPad Inc.). DFS were estimated by Kaplan–Meier method, with the p value determined by a log-rank test. The optimal cut-off point for outcome-based parameters was determined by plotting the probability parameter using the X-tile program (v3.6.1)^43^. The RNA-seq data from the Nanostring nCounter system were processed and analyzed using the R (v 3.6.3). The level of significance was set at 0.05.

### Reporting summary

Further information on research design is available in the Nature Research Reporting Summary linked to this article.

### Supplementary information

supplementary tables and figures

Reporting summary

## Data Availability

The datasets used and analyzed in this study are available from the corresponding author at reasonable request.

## Code availability

The authors are able to provide the codes supporting the findings of this study upon reasonable request.
