# Supplementary material for: Prognostic patterns in invasion lymph nodes of lung adenocarcinoma reveal distinct tumor microenvironments
Source: NPJ Precis Oncol. 2024 Jul 30;8:164. doi: 10.1038/s41698-024-00639-1 (PMC11289302; doi:10.1038/s41698-024-00639-1)
Supplement: Supplementary file 1 — supplementary tables and figures [file 41698_2024_639_MOESM1_ESM.pdf]

Supplementary Table 1. The NanoString nCounter 289 transcriptional Panel

ABCF1	CD44	FCGR1A	IL21R	NFKBIA	TIE1
ADM	CD47	FCGR2B	IL2RA	NKG7	TIGIT
ADORA2A	CD48	FCRL2	IL2RB	NOS2	TLR3
AKT1	CD6	FGF13	IL2RG	NT5E	TLR7
ANGPT2	CD68	FOXP3	IL4	OAS1	TLR8
ARG1	CD69	FPR1	IL6	OAS2	TLR9
ATM	CD70	FUT4	IL7R	OAS3	TNF
AXL	CD74	G6PD	IRF1	PDCD1	TNFRSF14
BCL2	CD79A	GBP1	IRF4	PDCD1LG2	TNFRSF17
BIRC5	CD79B	GNLY	IRF9	PDGFA	TNFRSF18
BLK	CD80	GUSB	ISG15	PDGFB	TNFRSF1A
BLM	CD84	GZMA	ITGA1	PECAM1	TNFRSF1B
BRCA1	CD86	GZMB	ITGAE	PIK3CA	TNFRSF4
BRCA2	CD8A	GZMH	ITGAL	PIK3CD	TNFRSF9
BRIP1	CD8B	GZMK	ITGAM	PMS2	TNFSF10
BTLA	CDKN2A	HAVCR2	ITGAX	PNOC	TNFSF13B
C1QA	CEACAM3	HDC	ITGB2	POLR2A	TNFSF18
C1QB	CMKLR1	HERC6	KIR2DL3	PRF1	TNFSF4
CCL13	CPA3	HIF1A	KIR3DL1	PSMB10	TNFSF9
CCL18	CSF1R	HLA-DMA	KIR3DL2	PSMB9	TRAT1
CCL2	CSF2	HLA-DMB	KLRB1	PTEN	TWIST1
CCL20	CSF2RB	HLA-DOA	KLRD1	PTGER4	VCAM1
CCL21	CSF3R	HLA-DOB	KLRK1	PTGS2	VEGFA
CCL22	CTAG1B	HLA-DPA1	LAG3	PTPN11	VTCN1
CCL4	CTLA4	HLA-DQA2	LCK	PTPRC	ZAP70
CCL5	CTSS	HLA-DRA	LILRB2	PVR	ZEB1
CCL7	CTSW	HSD11B1	LY9	RAD51	CXCL2
CCND1	CX3CL1	ICAM1	LYZ	RB1	FCGR3B
CCR2	CX3CR1	ICOS	MAGEA1	RORC	GZMM

CCR4	CXCL1	ICOSLG	MAGEA12	RUNX3	HLA-DQA1
CCR5	CXCL10	IDO1	MAGEA4	S100A12	HLA-DRB1
CD14	CXCL11	IFI27	MAGEC2	S100A8	HLA-E
CD163	CXCL12	IFI35	MELK	S100A9	OAZ1
CD19	CXCL13	IFI6	MKI67	SDHA	PF4
CD1C	CXCL5	IFIH1	MLANA	SELL	PRR5
CD2	CXCL8	IFIT1	MLH1	SH2D1A	STK11IP
CD209	CXCL9	IFIT2	MMP9	SIGLEC5	TBC1D10B
CD244	CXCR2	IFIT3	MRC1	SLAMF7	TPSAB1
CD247	CXCR3	IFITM1	MS4A1	SNAI1	UBB
CD27	CXCR4	IFITM2	MS4A2	SPIB	
CD274	CXCR6	IFNG	MS4A4A	STAT1	
CD276	CYBB	IL10	MSH2	STAT3	
CD28	DLL4	IL10RA	MSH6	STAT4	
CD38	EGFR	IL12RB2	MTOR	TAP1	
CD3D	EIF2AK2	IL15	MX1	TBP	
CD3E	ENTPD1	IL17A	MYC	TBX21	
CD3G	EOMES	IL18	NBN	TCL1A	
CD4	FAS	IL1A	NCAM1	TDO2	
CD40	FASLG	IL1B	NCR1	TFRC	
CD40LG	FCAR	IL2	NECTIN2	TGFB1	

Supplementary Table 2. RNA extraction information of TDLNs+ tissue pathological section

Pathological section number	Encoding	TDLNs+ patterns	RNA (ng/μl)
1802381-2	A01	Polarized	1846.8
1809551-2	A02	Polarized	2057
1810170-9	A03	Polarized	1238
1810833-4	A04	Polarized	2029.2
1821961-7	A05	Polarized	#N/A
1901348-8	A06	Polarized	684
1906782-11	A07	Polarized	2196.4
1907078-3	A08	Polarized	2242
1909792-10	A09	Polarized	1976
1910900-2	A10	Polarized	1839.2
1611351-3	F01	Scattered	1573.2
1617894-8	F02	Scattered	2454.8
1712908-11	F03	Scattered	3040
1717097-3	F04	Scattered	2249.6
1801655-10	F05	Scattered	1786
1813567-2	F06	Scattered	592.8
1903680-7	F07	Scattered	1444
1905790-5	F08	Scattered	1307.2
1910165-5	F09	Scattered	2644.8
1708465-9	F10	Scattered	1466.8
1705414-9	B01	Colloid	#N/A
1818286-8	B02	Colloid	2097.6
1819199-17	B03	Colloid	1862
1902566-6	B04	Colloid	2325.6
1905002-9	B05	Colloid	1786
1907768-5	B06	Colloid	2021.6
1703231-1	B07	Colloid	874
1713273-15	B08	Colloid	2082.4
1803414-7	B09	Colloid	2470
1805894-9	B10	Colloid	3252.8
1705775-2	C01	Necrosis	2135.6
1706259-7	C02	Necrosis	2302.8
1718326-4	C03	Necrosis	2523.2
1815572-2	C04	Necrosis	2675.2
1816365-10	C05	Necrosis	1109.6
1816452-6	C06	Necrosis	2166
1902946-9	C07	Necrosis	1580.8
1904798-6	C08	Necrosis	2538.4
1912062-13	C09	Necrosis	1694.8
1913069-2	C10	Necrosis	3754

1618400-12	D01	Specific	#N/A
1618798-1	D02	Specific	1307.2
1618951-9	D03	Specific	2097.6
1700025-4	D04	Specific	2242
1717376-6	D05	Specific	1816.4
1801942-11	D06	Specific	#N/A
1803491-7	D07	Specific	1299.6
1806686-8	D08	Specific	2432
1818454-16	D09	Specific	1694.8
1717187-11	D10	Specific	2325.6
1900717-10	E01	Common	1276.8
1616147-16	E02	Common	#N/A
1902652-13	E03	Common	2401.6
1902882-16	E04	Common	1618.8
1904694-4	E05	Common	896.8
1904692-5	E06	Common	311.6
1907623-5	E07	Common	2234.4
1904098-9	E08	Common	1710
1908578-14	E09	Common	#N/A
1909522-7	E10	Common	1512.4

#N/A: failed in the RNA extraction.

Supplementary Table 3. List of antibodies and corresponding metal isotope labels performed in IMC analysis

Target marker	Label	Source	Catalog#	Dilution
CD19	142Nd	Fluidigm	3142014D	1:100
CD56	143Nd	Abcam	ab251595	1:75
CXCR5	144Nd	Abcam	ab272936	1:100
IFN-γ	145Nd	Abcam	ab218890	1:100
CD62L	146Nd	Proteintech	26477-1-AP	1:100
BCL6	147Sm	Fluidigm	3147020D	1:100
CXCL10	148Nd	Affinity	DF6417	1:100
CD8b	149Sm	Invitrogen	MA5-29134	1:100
PD-L1	150Nd	Fluidigm	3150031D	1:100
CD107a	151Eu	Fluidigm	3151021D	1:100
D2-40/PDPN	152Sm	Novusbio	NBP2-03952	1:50
CD44	153Eu	Fluidigm	3153029D	1:400
AID/AICDA	154Sm	Abcam	ab269457	1:100
Foxp3	155Gd	Fluidigm	3155016D	1:200
CD4	156Gd	Fluidigm	3156033D	1:200
CCL19	158Gd	Abcam	ab221704	1:200
CD68	159Tb	Fluidigm	3159035D	1:200
CCL21	160Gd	Abcam	ab89396	1:200
CD20	161Dy	Fluidigm	3161029D	1:200
CCR7	162Dy	Abcam	ab27293	1:200
CD24	163Dy	Abcam	ab199140	1:100
Cytokeratin 7	164Dy	Fluidigm	3164028D	1:200

PD-1	165Ho	Fluidigm	3165039D	1:100
CD74	166Er	Fluidigm	3166025D	1:100
CD31	167Er	Abcam	ab207090	1:200
Ki67	168Er	Fluidigm	3168022D	1:200
p40	169Tm	Abcam	ab269956	1:100
CD3	170Er	Fluidigm	3170019D	1:100
CD21	171Yb	Abcam	ab240987	1:100
FAP	172Yb	Abcam	ab53066	1:200
HIF1a	173Yb	Abcam	ab210073	1:200
LAMP3/CD63	174Yb	Abcam	ab215821	1:200
HLA-ABC	175Lu	Abcam	ab239788	1:200
TWIST1	176Yb	Millipore	ABD29	1:200

A

Scattered-type **A-E**(20x); **a-e**(200X)

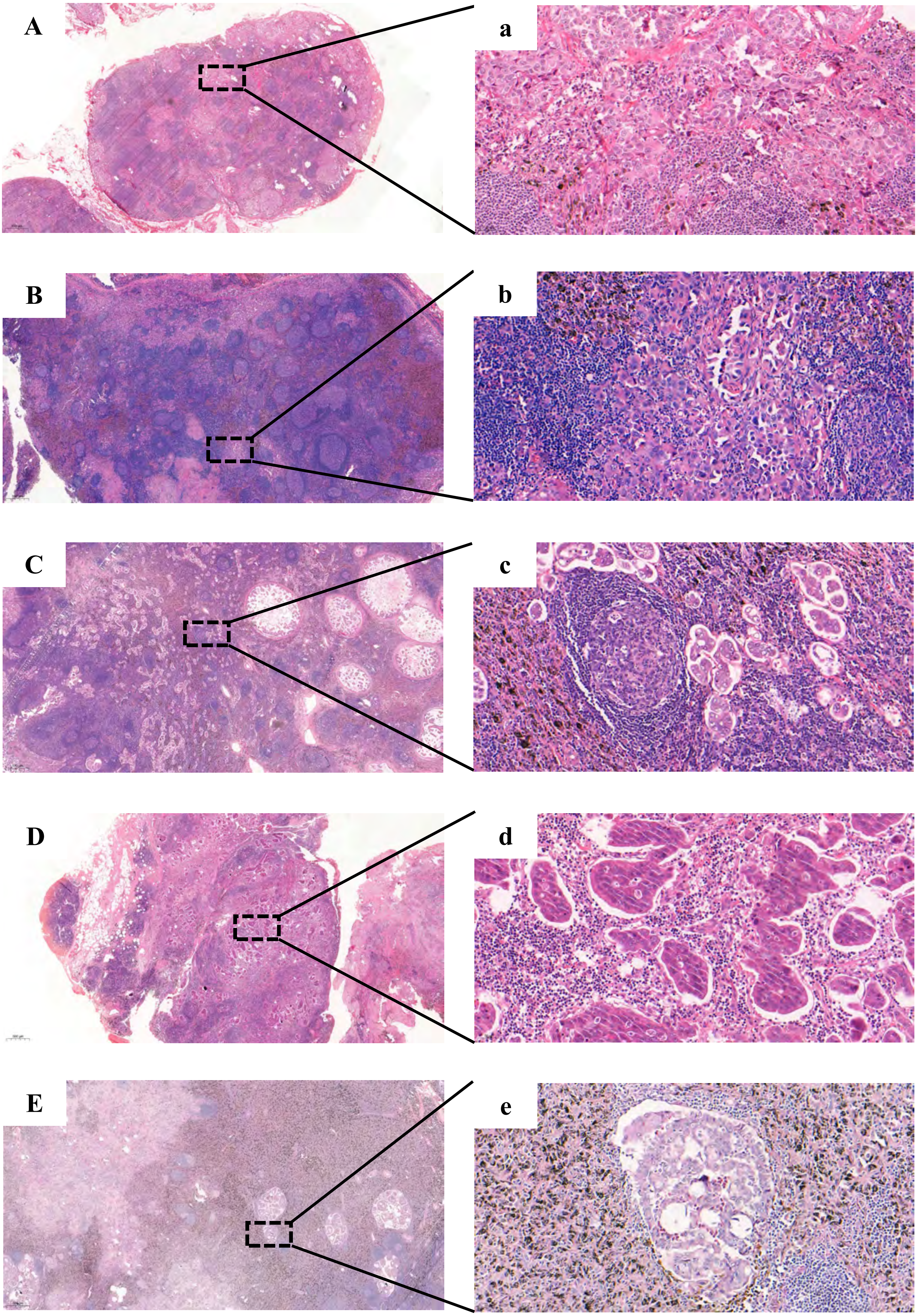

B Polarized-type **A-E**(20x); **a-e**(200X)

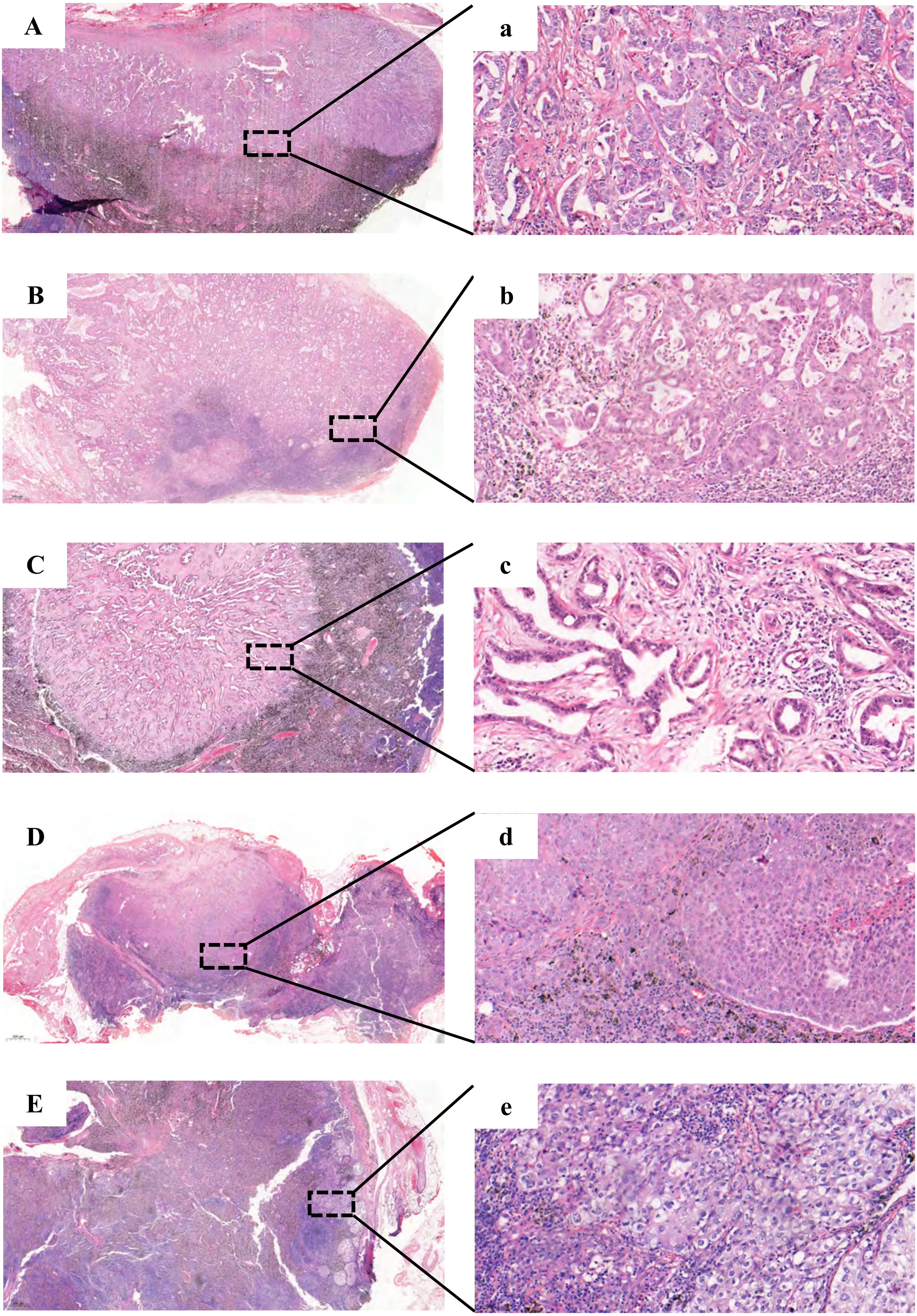

Supplementary Figure 1. The detailed information of the TDLNs+ polarized and scattered pattern in the cohort. Five cases for each TDLNs+ pattern type. Magnification:20x, 200X, respectively).

A

Colloid-type **A-E**(20x); **a-e**(200X)

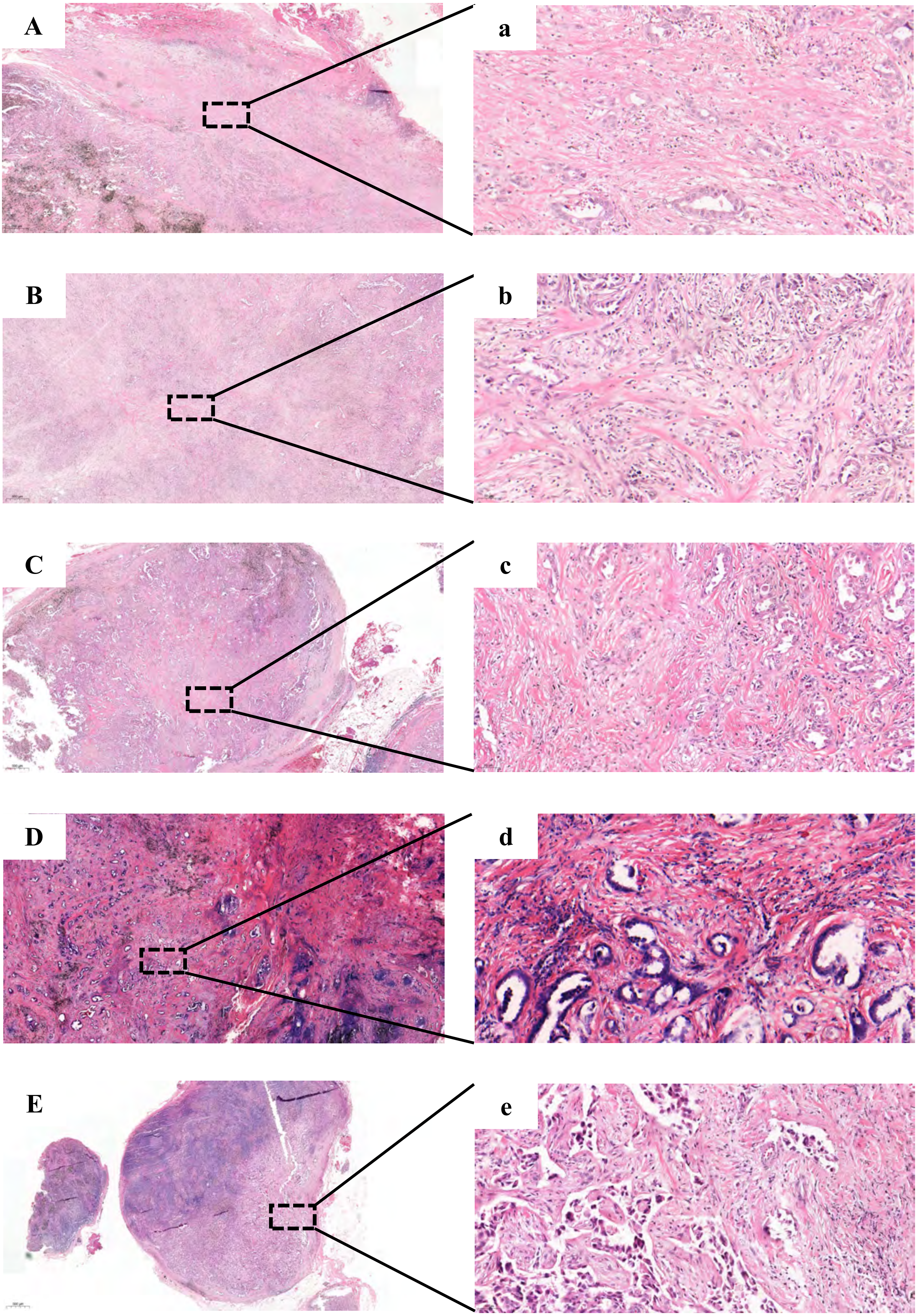

B Necrosis-type_tumor necrosis **A-E**(20x); **a-e**(200X)

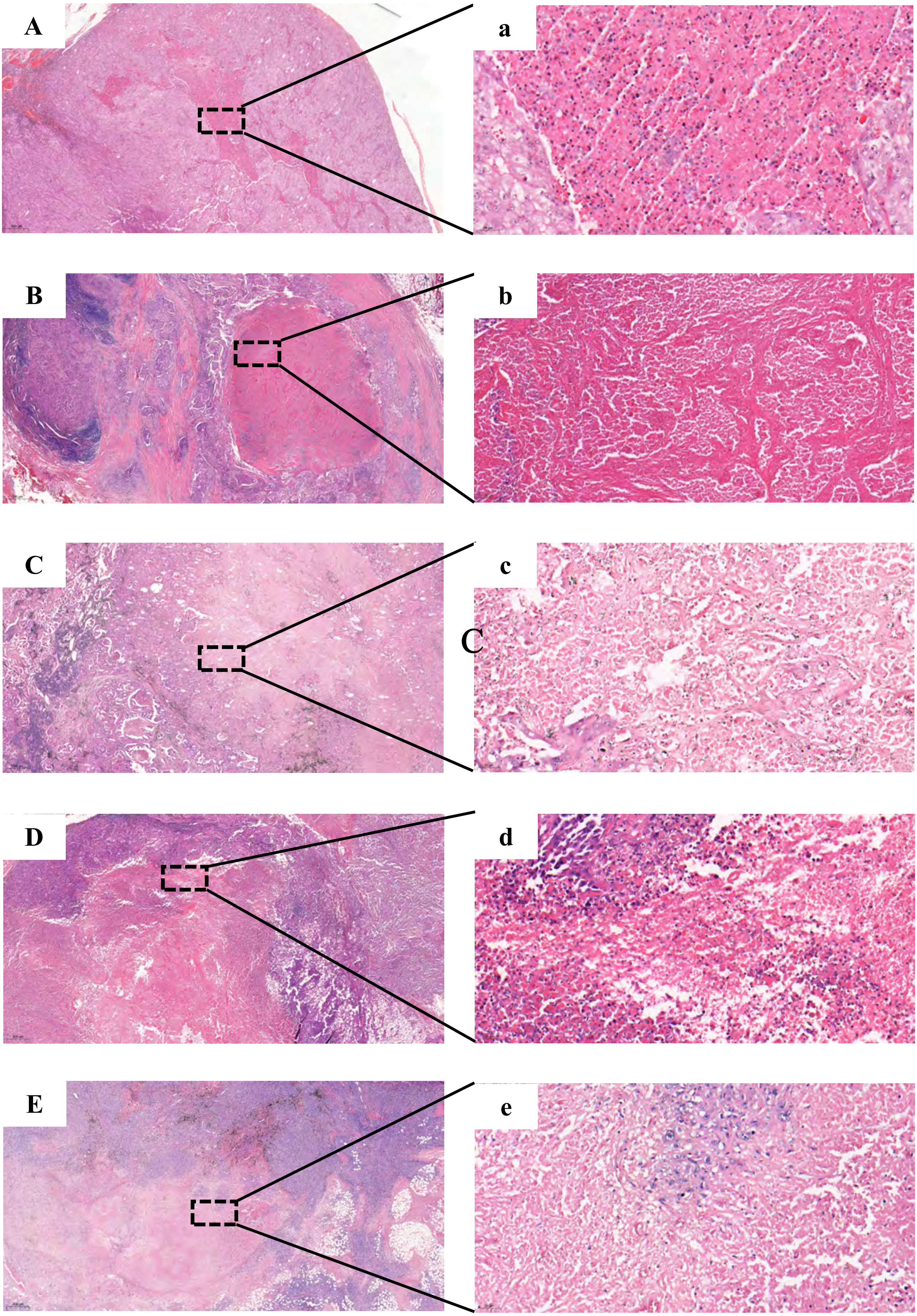

C Necrosis-type_hemorrhagic **A-E**(20x); **a-e**(200X)

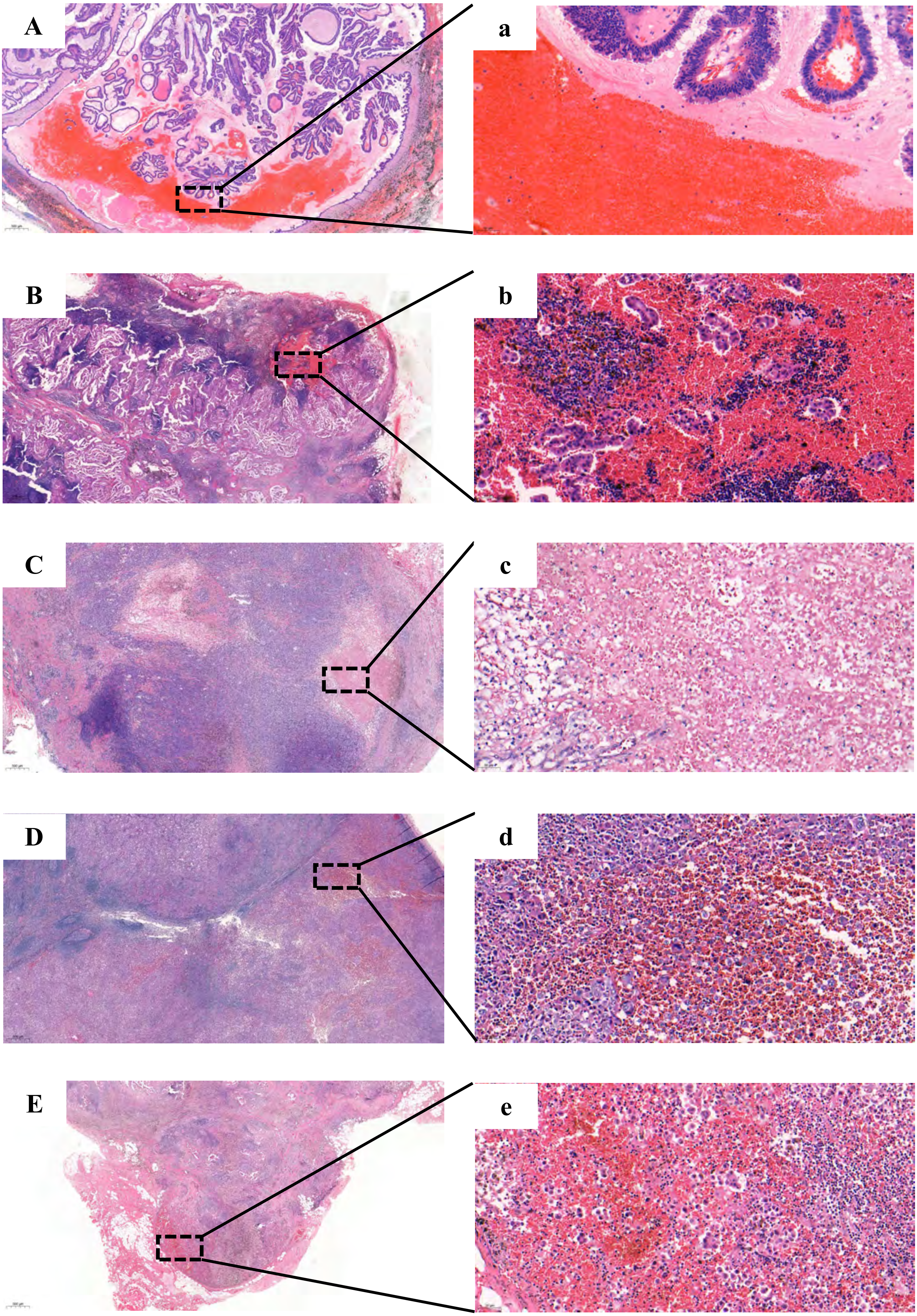

D Specific-type_psammoma bodies **A-E**(20x); **a-e**(200X)

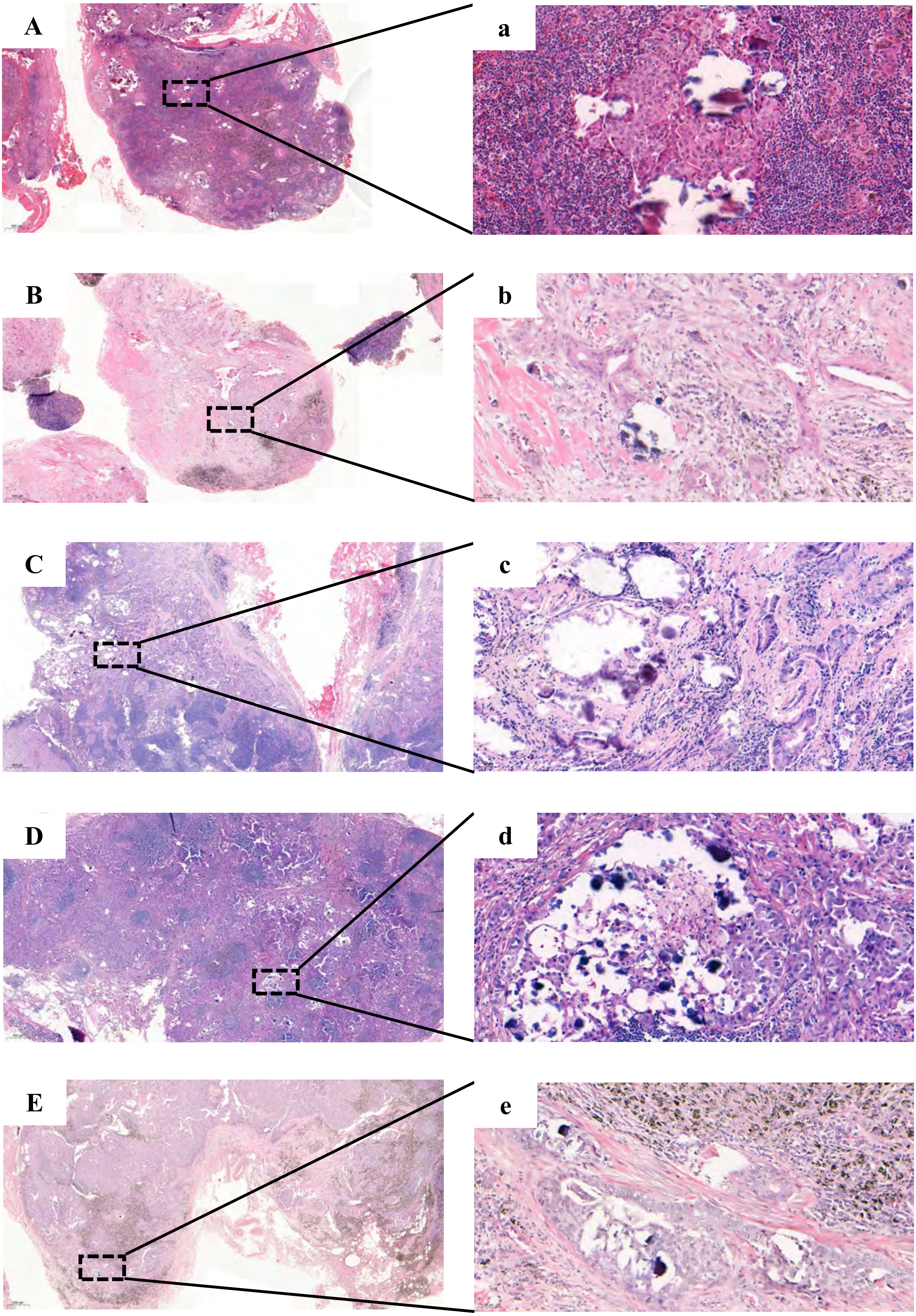

E Specific-type_hyalinosis **A-E**(20x); **a-e**(200X)

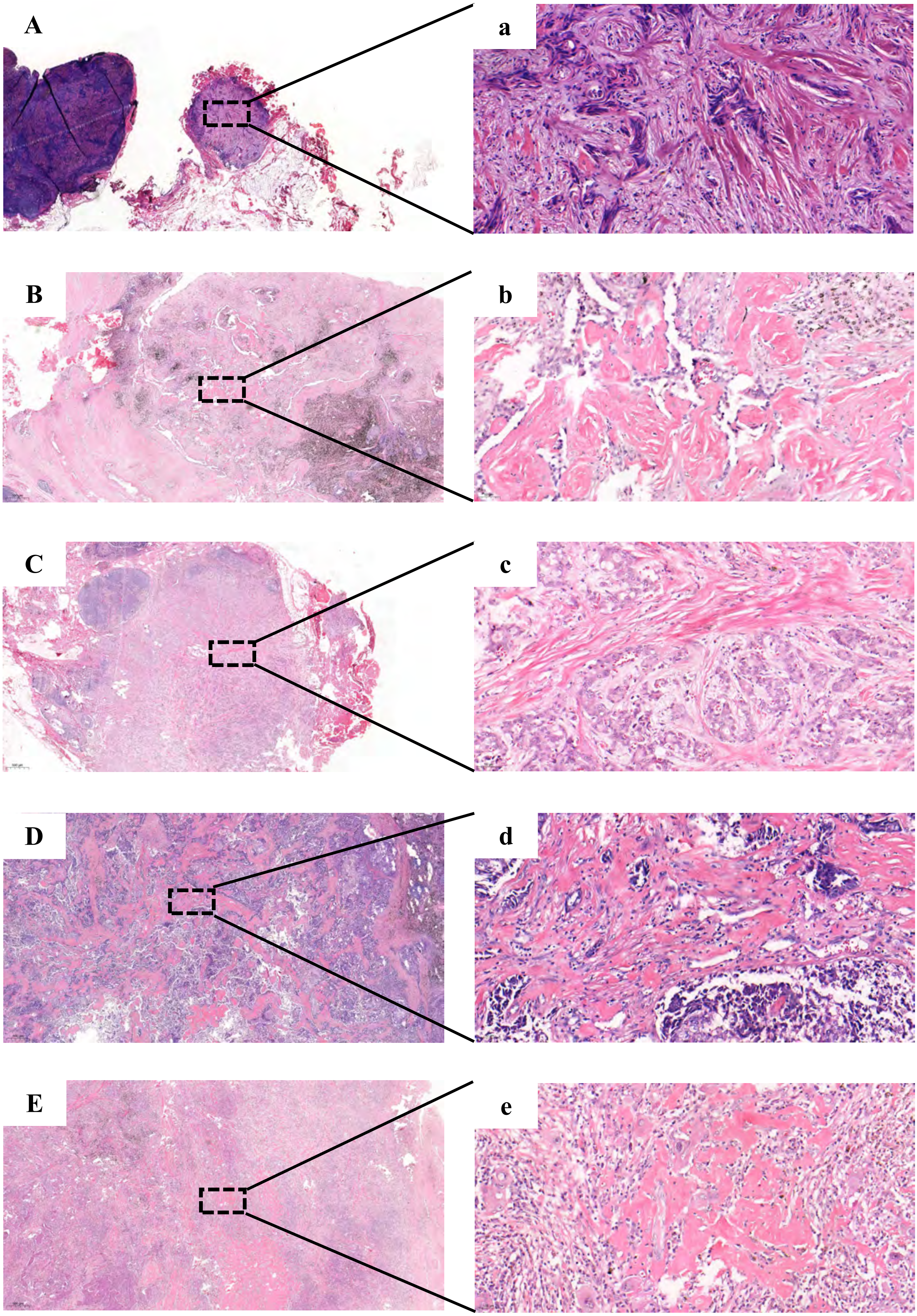

F Common-type **A-E**(20x); **a-e**(200X)

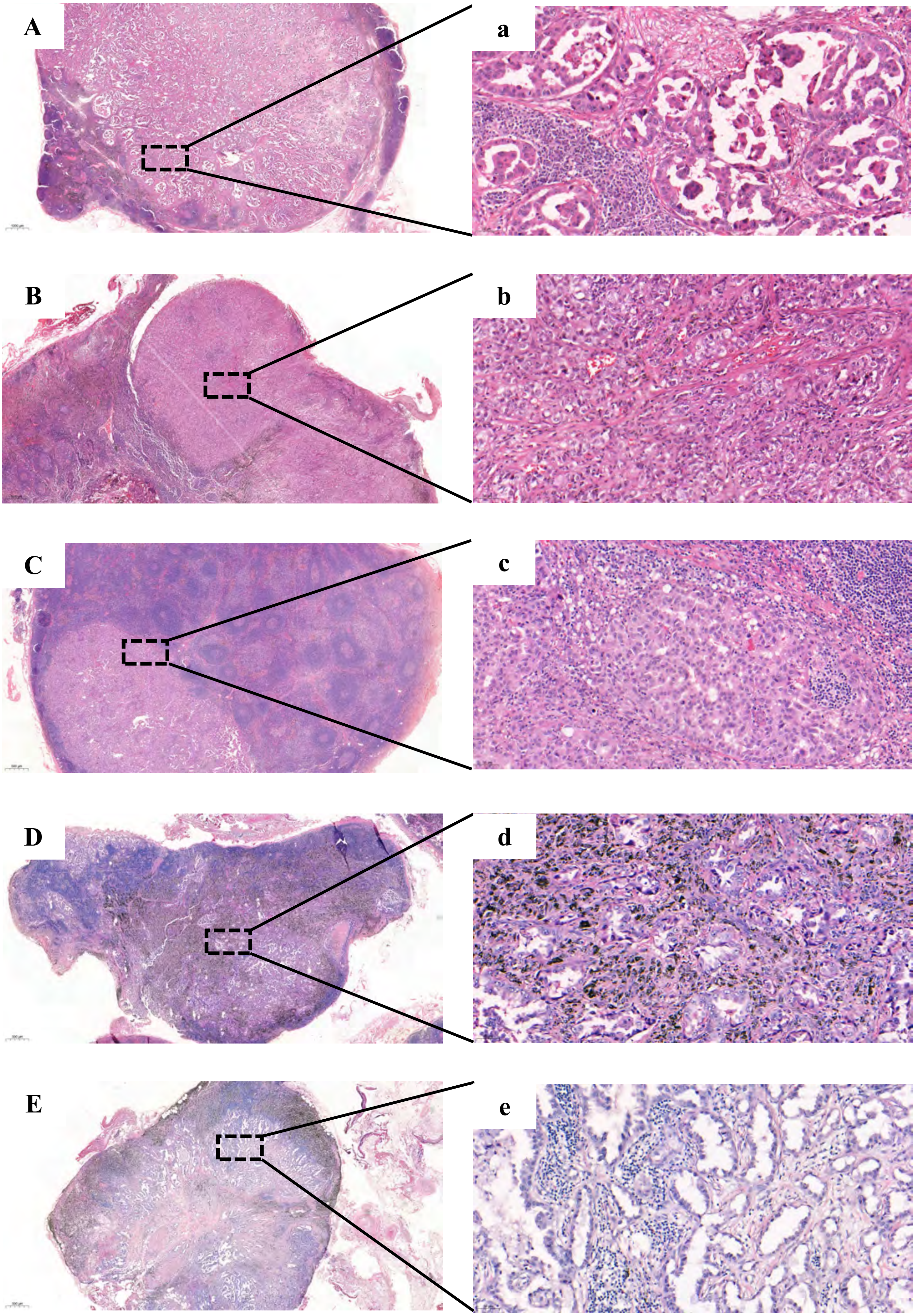

Supplementary Figure 2. The detailed information of the TDLNs+ common, necrosis, colloid, and specific patterns in the cohort. Five cases for each TDLNs+ pattern type. Magnification:20x, 200X, respectively).

Antigen	Description
**Tumor Context**	
Cytokeratin 7	Luminal CK
p40	Basal Marker
**Adhesion Molecules**	
CD24	Cell Adhesion
CD44	Cancer Stem Cell
**Immune Context**	
HLA_ABC	Pan-Immune
CD3	T Cells
CD4	T Cells
CD8b	T Cells
CD56	NK Cells
CD107a	^{activated}NK Cells
CD68	Macrophages
CD74	Dendritic cells(DCs)
CD63/LAMP3	^{immunesuppressive}DCs
Foxp3	Treg
IFNr	^{activated}Immune Cells
**Tumor Malignant Phenotype**	
TWIST1	EMT-related marker
HIF1-a	Hypoxia
KI67	Cell Growth

Antigen	Description
**Endothelial**	
CD31	Endothelium
PDPN	Lymphatic Endothelium
**Mesenchymal Markers**	
FAP	Fibroblast Cells
CD62L	HEV Cells
**Tertiary lymphoid structures**	
CD20	B Cells
CD21	follicular DCs (FDCs)
CD19	B Cells activation
AID	B Cells differentiation
BCL6	B Cells differentiation
**Cytokines/Chemokines & Receptors**	
CCL21	Pan-Immune attraction
CCL19	Pan-Immune attraction
CCR7	T/B Cells migration
CXCR5	B Cells migration
CXCL10	Monocytes migration
CXCL13	B Cells migration
**Immune Checkpoints**	
PD-1	Co-inhibitory marker
PD-L1	Co-inhibitory marker

Supplementary Figure 3. The 35-antibodies imaging mass cytometry (IMC) panel.

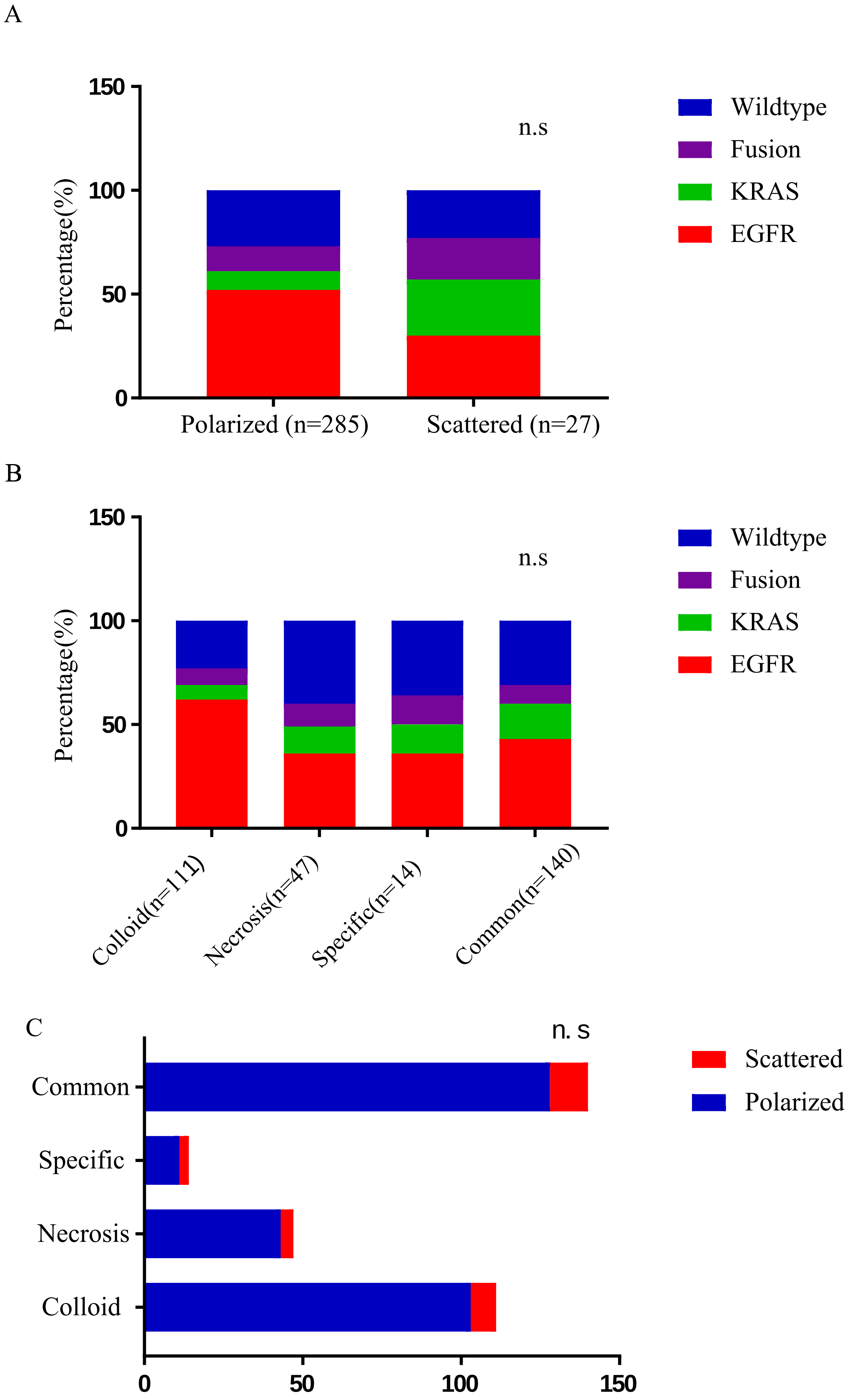

Supplementary Figure 4. Association between infiltrated TDLNs+ patterns and gene mutations. (A, B) LUAD driver mutations were compared between patients with different TDLNs+ patterns in the cohort (for detailed statistical methods, see Table 2 and Table 3). (C) Association between the tumor-infiltrated TDLNs+ pattern and the TME-compositional TDLNs + pattern, chi-square tests for categorical variables ($\chi 2 = 3.187$, $p = .364$).

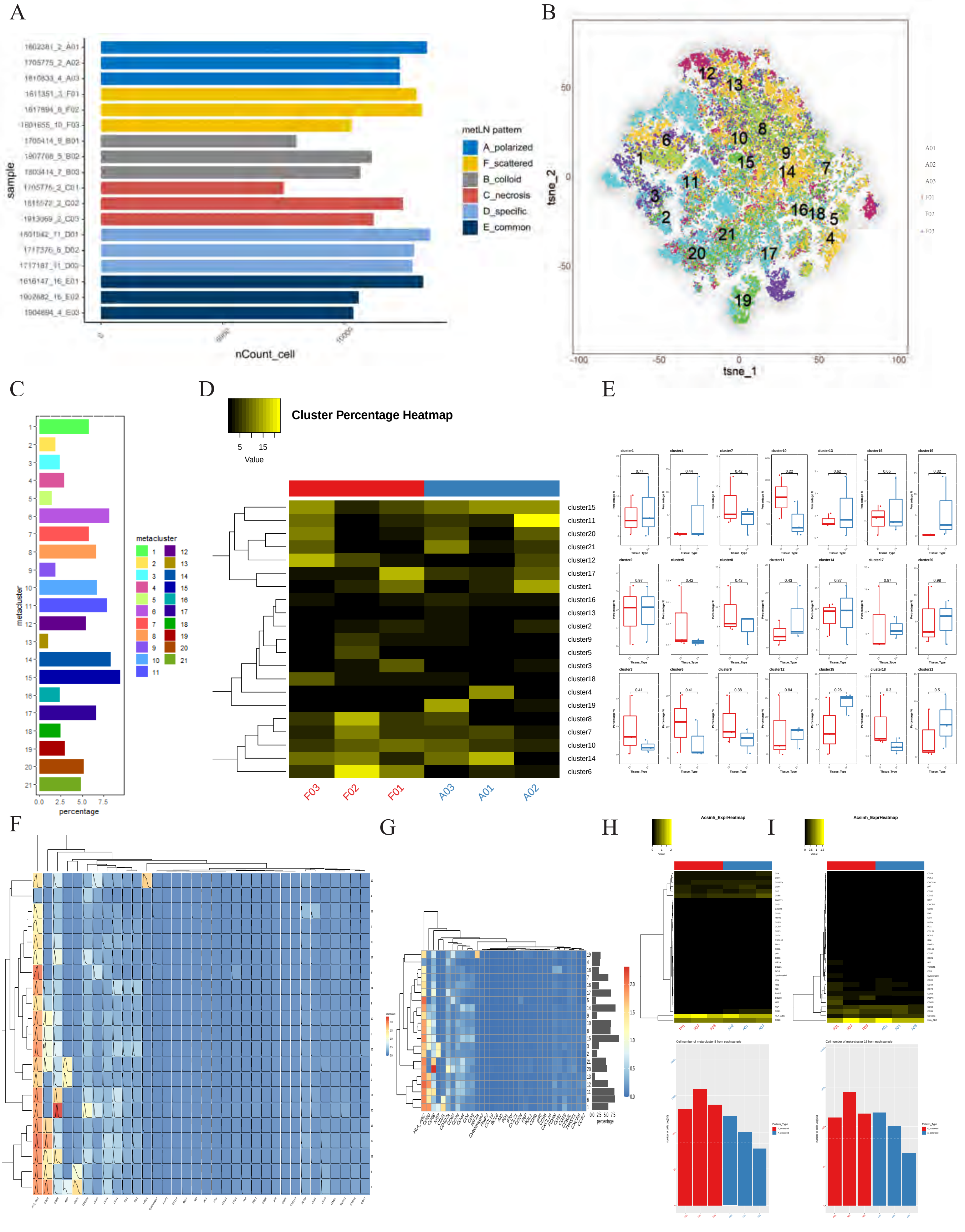

Supplementary Figure 5. IMC analysis of the tumor-infiltrated TDLN+ pattern. (A) The plot depicts the total number of cells detected in Regions of Interest (ROIs) from the TDLNs+ pattern (3 patients each). (B) tSNE-plot of the unsupervised clustering analysis from the tumor-infiltrated TDLNs+ pattern showing 21 distinct meta-clusters with varying expression levels across multiple phenotypes. (C) Stack bar plots of cell-type meta cluster densities. (D) Heat map demonstrating the proportions of meta cluster present within each patient. (E), (F) Abundance box and density plots depicting the average of each meta cluster in the tumor-infiltrated TDLNs+ pattern. (G) Heat map showing z-scored mean marker expression of the single-cell phenotypic cluster. (H), (I) Heat map and box plot indicating meta cluster 8 (HLA_ABCloCD20lo CD21lo) and meta cluster 18 (PDPNhiCD31hi) in predominantly scattered TDLNs+ pattern.

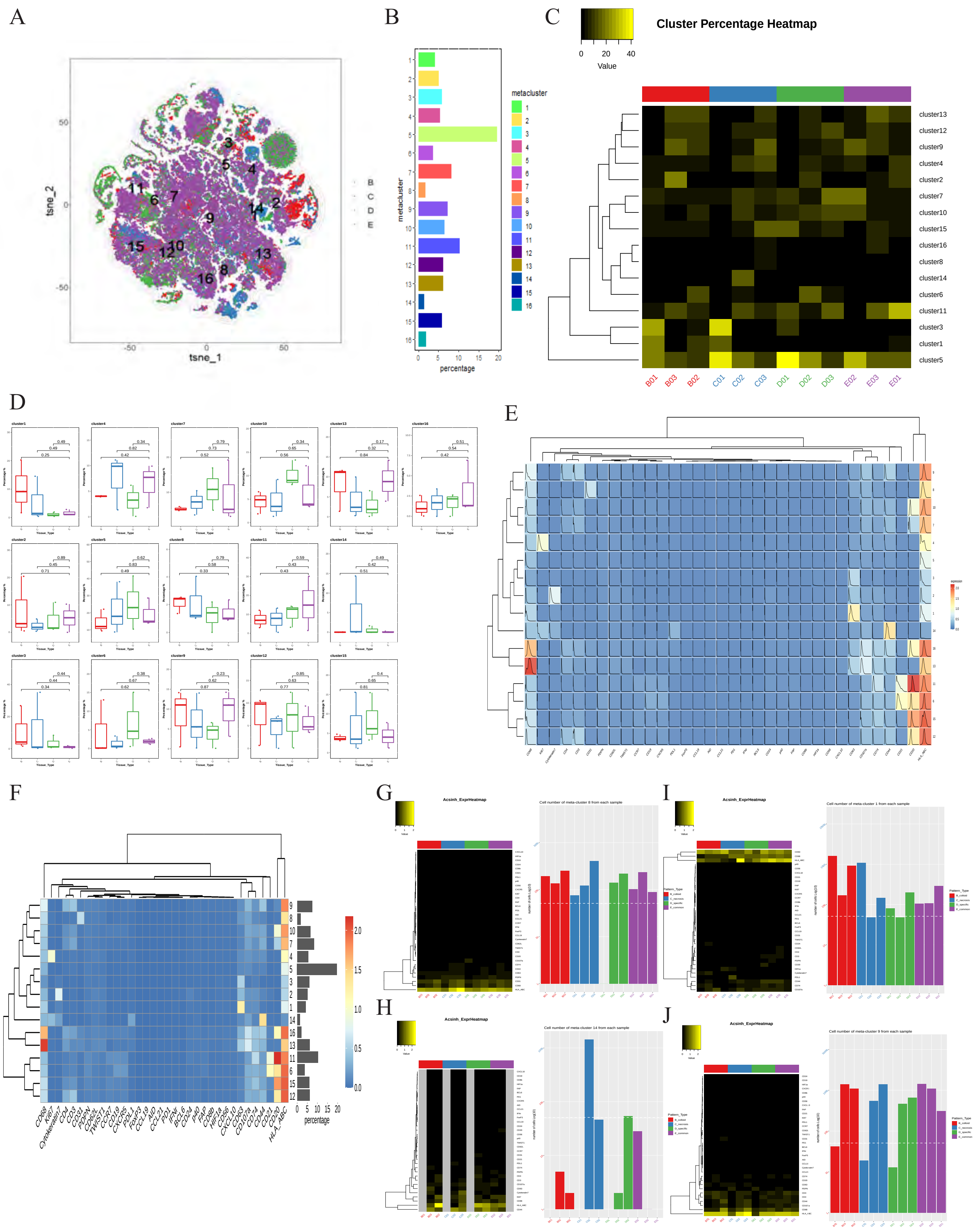

Supplementary Figure 6. IMC analysis of the TME-compositional TDLN+ pattern. (A) tSNE-plot of the unsupervised clustering analysis from the TME-compositional TDLNs+ pattern showing 16 distinct meta-clusters with varying expression levels across multiple phenotypes. (B) Stack bar plots of cell-type meta cluster densities. (C) Heat map demonstrating the proportions of meta cluster present within each patient. (D), (E) Abundance box and density plots depicting the average of each meta cluster in the TME-compositional TDLNs+ pattern. (F) Heat map showing z-scored mean marker expression of the single-cell phenotypic cluster. (G), (H) Heat map and box plot indicating a high cellular density of meta-cluster 8 (PDPNhiCD31hi) and meta-cluster 14 (CK7hiCD44hiHLA_ABClo) in necrosis TDLNs+ pattern. (I) Heat map and box plot showing a group of suspected meta cluster 1 anergic antigen-presenting cells (CD63/Lamp3hiCD68loHLA_ABClo) in the colloid subtype. (J) Heat map and box plot revealing larger densities of activated immune cells meta cluster 9 (HLA_ABChiCD3hiCD4hiCD68hiCD107ahi).
