# Supplementary material for: Prognostic patterns in invasion lymph nodes of lung adenocarcinoma reveal distinct tumor microenvironments
Source: NPJ Precis Oncol. 2024 Jul 30;8:164. doi: 10.1038/s41698-024-00639-1 (PMC11289302; doi:10.1038/s41698-024-00639-1)
Supplement: Supplementary file 2 — Reporting summary [file 41698_2024_639_MOESM2_ESM.pdf]

nature portfolio

Corresponding author(s): Wenhua Liang

Last updated by author(s): Feb 6, 2024

Reporting Summary

Nature Portfolio wishes to improve the reproducibility of the work that we publish. This form provides structure for consistency and transparency in reporting. For further information on Nature Portfolio policies, see our Editorial Policies and the Editorial Policy Checklist.

Statistics

For all statistical analyses, confirm that the following items are present in the figure legend, table legend, main text, or Methods section.

n/a	Confirmed	
☐	☒	The exact sample size (*n*) for each experimental group/condition, given as a discrete number and unit of measurement
☐	☒	A statement on whether measurements were taken from distinct samples or whether the same sample was measured repeatedly
☐	☒	The statistical test(s) used AND whether they are one- or two-sided<br>*Only common tests should be described solely by name; describe more complex techniques in the Methods section.*
☒	☐	A description of all covariates tested
☐	☒	A description of any assumptions or corrections, such as tests of normality and adjustment for multiple comparisons
☐	☒	A full description of the statistical parameters including central tendency (e.g. means) or other basic estimates (e.g. regression coefficient) AND variation (e.g. standard deviation) or associated estimates of uncertainty (e.g. confidence intervals)
☐	☒	For null hypothesis testing, the test statistic (e.g. *F*, *t*, *r*) with confidence intervals, effect sizes, degrees of freedom and *P* value noted<br>*Give P values as exact values whenever suitable.*
☒	☐	For Bayesian analysis, information on the choice of priors and Markov chain Monte Carlo settings
☒	☐	For hierarchical and complex designs, identification of the appropriate level for tests and full reporting of outcomes
☒	☐	Estimates of effect sizes (e.g. Cohen's *d*, Pearson's *r*), indicating how they were calculated

Our web collection on statistics for biologists contains articles on many of the points above.

Software and code

Policy information about availability of computer code

Data collection	NA.
Data analysis	The following software was used in this study:<br>R (v3.6.3), CaseViewer (v2.2), NanoStringDiff, nSolver (v2.6), Clusterprofiler (v4.0), ggplot2, MCDTM viewer, CellProfiler (v4.1.3), histoCAT (v1.7.6), Phenograph (v0.99.1), Rtsne (v.0.15), SPSS (v26.0), Prism (v7.0), and X-tile program (v3.6.1).

For manuscripts utilizing custom algorithms or software that are central to the research but not yet described in published literature, software must be made available to editors and reviewers. We strongly encourage code deposition in a community repository (e.g. GitHub). See the Nature Portfolio guidelines for submitting code & software for further information.

Data

Policy information about availability of data

All manuscripts must include a data availability statement. This statement should provide the following information, where applicable:

- Accession codes, unique identifiers, or web links for publicly available datasets
- A description of any restrictions on data availability
- For clinical datasets or third party data, please ensure that the statement adheres to our policy

All relevant data are available from the authors on reasonable request.

Research involving human participants, their data, or biological material

Policy information about studies with human participants or human data. See also policy information about sex, gender (identity/presentation), and sexual orientation and race, ethnicity and racism.

Reporting on sex and gender	Information of patient sex was provided in the Table 1.
Reporting on race, ethnicity, or other socially relevant groupings	None of such information was collected in this study.
Population characteristics	Population of patient age, smoke, driver mutation, tumor size, vascular invasion, N stage, DFS status, primary tumor WHO classification, metastatic WHO classification, infiltrated pattern,and compositional pattern.
Recruitment	Lung adenocarcinoma patients in the first affiliated hospital of Guangzhou Medical University of Thoracic Surgery and Oncology department preparing for surgery consented to collect bio-specimens before surgery. Patients who were diagnosed with lung adenocarcinoma and agreed to sign the consent file were retrospectively recruited in our study without any self-selection.
Ethics oversight	This retrospective study adheres to pertinent ethical guidelines and received approval from the Ethics Committee of the First Affiliated Hospital of Guangzhou Medical University, with consent waived.

Note that full information on the approval of the study protocol must also be provided in the manuscript.

Field-specific reporting

Please select the one below that is the best fit for your research. If you are not sure, read the appropriate sections before making your selection.

☒ Life sciences ☐ Behavioural & social sciences ☐ Ecological, evolutionary & environmental sciences

For a reference copy of the document with all sections, see nature.com/documents/nr-reporting-summary-flat.pdf

Life sciences study design

All studies must disclose on these points even when the disclosure is negative.

Sample size	No statistacal methods were used to pre-determine the sample size. The sample size of the tumor-invaded TDLNs (TDLNs+) was based on the number of lung adenocarcinoma samples available from the first affiliated hospital of Guangzhou Medical University.
Data exclusions	In the RNA extraction process, the polarized pattern failed in 1 case, the colloid failed in 1 case, the specific pattern failed in 2 cases, and the common pattern failed in 2 cases, and these samples were excluded from the Nanostring transcriptional profiling analysis.
Replication	The examination of the tumor-infiltrated pattern revealed that 285 patients demonstrated the polarized-type pattern, while 27 patients exhibited the scattered-type pattern. An analysis of the TME-composition pattern indicated that 140 patients showed the common-type pattern, 111 patients displayed the colloid-type pattern, 47 patients demonstrated the necrosis-type pattern, and 14 patients showed the specific-type pattern. As for Nanostring transcriptional analysis of the tumor-immune microenvironment, N=10 for each group; however, in the RNA extraction process, the polarized pattern failed in 1 case, the colloid failed in 1 case, the specific pattern failed in 2 cases, and the common pattern failed in 2 cases, and these samples were excluded. Moreover, IMC analysis of the tumor-infiltrated and TME-compositional TDLNs+ pattern (n=3 for each group).
Randomization	Randomization is not applicable in this study.
Blinding	There is no intervention to patients recruited, and it's not a clinical trail. Blinding is not applicable.

Reporting for specific materials, systems and methods

We require information from authors about some types of materials, experimental systems and methods used in many studies. Here, indicate whether each material, system or method listed is relevant to your study. If you are not sure if a list item applies to your research, read the appropriate section before selecting a response.

Materials & experimental systems

n/a	Involved in the study
☒	☐ Antibodies
☒	☐ Eukaryotic cell lines
☒	☐ Palaeontology and archaeology
☒	☐ Animals and other organisms
☐	☒ Clinical data
☒	☐ Dual use research of concern
☒	☐ Plants

Methods

n/a	Involved in the study
☒	☐ ChIP-seq
☒	☐ Flow cytometry
☒	☐ MRI-based neuroimaging

Clinical data

Policy information about clinical studies

All manuscripts should comply with the ICMJE guidelines for publication of clinical research and a completed CONSORT checklist must be included with all submissions.

Clinical trial registration

This is a retrospective study, and clinical trial registration is not applicable.

Study protocol

NA.

Data collection

We conducted a retrospective study of 312 lung adenocarcinoma patients who underwent R0 resection between 2015 and 2019 at the First Affiliated Hospital of Guangzhou Medical University.

Outcomes

We conducted a retrospective study of 312 LUAD patients who underwent R0 resection between 2015 and 2019 at the First Affiliated Hospital of Guangzhou Medical University. Clinicopathological data were retrieved from electronic records. In the cohort's median follow-up period of 22.5 months, there were 79 documented cases of recurrence. Analysis of the disease-free survival (DFS) of tumor-infiltrated and TME-composition TDLNs+ pattern.

Plants

Seed stocks

Report on the source of all seed stocks or other plant material used. If applicable, state the seed stock centre and catalogue number. If plant specimens were collected from the field, describe the collection location, date and sampling procedures.

Novel plant genotypes

Describe the methods by which all novel plant genotypes were produced. This includes those generated by transgenic approaches, gene editing, chemical/radiation-based mutagenesis and hybridization. For transgenic lines, describe the transformation method, the number of independent lines analyzed and the generation upon which experiments were performed. For gene-edited lines, describe the editor used, the endogenous sequence targeted for editing, the targeting guide RNA sequence (if applicable) and how the editor was applied.

Authentication

Describe any authentication procedures for each seed stock used or novel genotype generated. Describe any experiments used to assess the effect of a mutation and, where applicable, how potential secondary effects (e.g. second site T-DNA insertions, mosiacism, off-target gene editing) were examined.
